# miR-181a-5p mediates the effects of BMP4 on intestinal cell proliferation and differentiation

**DOI:** 10.1038/s41419-025-07730-w

**Published:** 2025-05-28

**Authors:** Chang Li, Yuning Zhou, Zhijie Yin, Yinping Jiang, Jinpeng Liu, Heidi L. Weiss, Qingding Wang, B. Mark Evers

**Affiliations:** 1 Markey Cancer Center, University of Kentucky, Lexington, KY USA; 2 Department of Surgery, University of Kentucky, Lexington, KY USA

**Keywords:** Colon cancer, Cell signalling

## Abstract

The intestinal mucosa undergoes a dynamic process of continual proliferation, differentiation, and apoptosis. Delineating the mechanisms involved in intestinal epithelial cell (IEC) differentiation is crucial to our understanding of not only normal gut adaptation but also aberrant intestinal growth. Bone morphogenetic protein (BMP) signaling is a pivotal regulator of intestinal proliferation and differentiation. However, the molecular underpinnings of the BMP pathway in this context are not entirely known. Here, we show a key role for the BMP4/microRNA (miR)-181/glycolysis signaling pathway in the maintenance of intestinal epithelial cell proliferation and differentiation. Treatment with BMP4 increased the expression of enterocyte markers and decreased proliferation of IECs, and importantly, decreased the expression of miR-181a-5p in mouse and human intestinal organoids. miR-181a-5p is a member of the miR-181 family with the highest expression in IECs. Treatment with locked nucleic acid (LNA) miR-181a-5p inhibitor significantly increased enterocyte differentiation as noted by increased expression of enterocyte markers in human and mouse intestinal organoids. In addition, LNA miR-181a-5p inhibitor repressed intestinal stem cell self-renewal as noted by the decreased organoid forming efficiency and expression of Ki67, cyclin D1, OLFM4 in human and mouse intestinal organoids. Moreover, in vivo administration of LNA miR-181a-5p inhibitor enhanced increased intestinal enterocyte differentiation and repressed intestinal cell proliferation. In contrast, overexpression of miR-181a-5p mimic decreased basal and BMP4-induced expression of enterocyte markers. Moreover, BMP4 treatment or inhibition of miR-181a-5p repressed hexokinase (HK) 1 expression and inhibited glycolysis. Consistently, knockdown of HK1 or inhibition of glycolysis using 2-deoxyglucose (2-DG) promoted enterocyte maturation and inhibited proliferation of IECs. Together, we provide evidence showing that miR-181a-5p inhibits intestinal enterocyte differentiation and promotes IEC proliferation through HK1-dependent glycolysis. Importantly, our findings identify miR-181a-5p as downstream in mediating BMP4 induction of enterocyte differentiation and inhibition of proliferation in IECs.

## Introduction

The intestinal epithelium consists of a multitude of self-renewing crypt-villus units that undergo a process of constant and rapid renewal. The intestinal crypts contain a highly dynamic niche with multipotent intestinal stem cells (ISCs) located at the base of the crypt, generating new cells that eventually differentiate into the specialized cell types with distinct functions [1, 2]. The differentiated enterocytes, which comprise the majority of the cells of the intestinal mucosa, undergo a process of apoptosis and are extruded into the lumen [1, 3]. Dysregulation of intestinal epithelial cell (IEC) proliferation and differentiation within the intestinal crypts is associated with several intestinal pathologies including colorectal cancer, inflammatory bowel disease (IBD), and necrotizing enterocolitis [4–6]. To date, the cellular mechanisms regulating ISC maintenance, proliferation, and differentiation are not entirely known.

ISCs are supported by the ISC niche, which produces opposing gradients of Wnt and bone morphogenetic protein (BMP) signaling that balance ISC self-renewal and differentiation. BMPs, mainly produced by intestinal mesenchymal stromal cells, are critical for the maintenance of intestinal homeostasis [7]. BMPs perform their signaling through the type IA receptor (BMPR1a) [8, 9]. BMP4, one of the most abundant BMP ligands in the intestine, restricts ISC self-renewal [10], triggers the differentiation of enterocytes, and contributes to the maintenance of homeostatic renewal of the intestinal epithelium [8]. However, how BMPs exert their functions and the downstream factors mediating the effects of BMPs in the intestine are not known [11].

MicroRNAs (miRNAs) are small non-coding RNAs that negatively regulate the levels of conserved target genes by directly binding to the 3′ untranslated regions. miRNAs are essential post-transcriptional repressors of mRNA targets regulating fundamental processes of cell differentiation, proliferation, development, and gut homeostasis [12, 13]. The miR-181 family, downregulated in the intestinal epithelium from patients with IBD, is required for protection against severe colonic inflammation in response to epithelial injury in mice [14].

Metabolic changes play a critical role in determining whether a cell remains quiescent, proliferates, or differentiates [15]. Tissue- and cell-specific metabolic pathways are tightly regulated during development and perform unique functions in specific cell contexts [15]. Proliferative cells at the base of the intestinal crypt are characterized by a glycolytic metabolic phenotype [16]. In contrast, energy generation in the differentiated enterocytes of the upper crypt mainly depend on mitochondrial oxidative phosphorylation [17]. Recently, we demonstrated that hexokinase 2 (HK2), a major contributor to increased glycolysis [18], acts downstream of Wnt/β-catenin signaling and is important for maintenance of intestinal homeostasis [19]. However, the signaling pathways and molecules regulating the metabolism in IECs are not well defined.

In this study, we describe a novel role for miR-181a-5p, acting downstream of BMP4, in the maintenance of intestinal homeostasis. We demonstrate that BMP4 represses the expression of miR-181a-5p, which regulates glycolysis, proliferation, and differentiation.

## Materials and methods

### Mouse intestinal crypt isolation and organoid culture

Intestinal crypts were isolated as previously described [20]. Briefly, mouse small intestine (SI) was opened longitudinally, washed with ice-cold PBS to remove the luminal contents, and cut into 2–4 mm pieces. The intestinal fragments were incubated in ice-cold PBS containing 10 mM EDTA for 60 min at 4 °C. Crypts were released by shaking with ice-cold PBS. Washing in ice-cold PBS was repeated until most of the crypts were released, as determined by microscopic analysis. Crypt suspensions were passed through a 70 μm cell strainer and centrifuged at 300 × g for 5 min. Isolated crypts were mixed with Matrigel (Corning #356231; Corning, NY) and cultured in IntestiCult™ Organoid Growth Medium (Stemcell Technologies, #06005; Vancouver, Canada) as described previously [21]. To determine the role of miR-181a-5p, mouse SI organoids were incubated with either miR-181a-5p miRCURY LNA miRNA inhibitor (catalog number: YCI0200407-FZA, Qiagen, Germantown, MD) or miRNA negative control oligos (catalog number: YCI0201861-FZA, Qiagen).

To determine the role of HK1, mouse SI organoids were infected with lentivirus expressing the HK1 shRNA or non-targeting control (NTC) shRNA (Sigma, St. Louis, MO). To determine the role of miR-181a-5p in the intestine, mouse SI organoids were infected with lentivirus expressing miR-181a-5p mimic (MLMIR0085, Sigma) or miRNA mimic control (NCLMIR002, Sigma). The infected organoids were cultured in the presence of puromycin (2 μg/ml) to select for organoid cells with stable knockdown of HK1 or stable overexpression of miR-181a-5p as described [22].

The organoid area was measured as described by Sorrentino et al. [23]. Briefly, the surface area of organoids was measured by taking several random nonoverlapping photos of organoids using a microscope. Photos were analyzed using ImageJ software. Organoid perimeters for area measurements were defined manually.

### In vivo miR-181a-5p inhibitor treatment in mice

C57BL/6 mice, purchased from the Jackson Laboratory (Sacramento, CA), were maintained on a 12 h light/dark schedule in filter top isolators with autoclaved water under specific pathogen-free conditions. All animal procedures were conducted with approval from and in compliance with the University of Kentucky Institutional Animal Care and Use Committee. Mice were randomly assigned to each group. Custom large scale in vivo miRCURY LNA inhibitors were used (Qiagen). These specific LNA inhibitors bind to the target miRNA, inhibiting RNA-Induced Silencing Complex loading. The control oligos and miR-181a-5p inhibitor were diluted in sterile PBS. C57BL/6 male mice (8–10 wks old) were injected intraperitoneally (IP) with miR-181a-5p miRCURY LNA miRNA inhibitor (20 mg/kg) or miRCURY LNA control oligos (20 mg/kg) as described [24]. Mice were sacrificed at 7 d post-injection. At sacrifice, intestines were harvested and proliferation, differentiation, and levels of miR-181a-5p was determined as described [19, 20].

### Human duodenum IEC isolation and organoid culture

Histologically normal human duodenal samples were obtained from patients at the University of Kentucky undergoing pancreatic resection for cancer. Collection of all patient materials for this study was obtained following patient consent and approved by the University of Kentucky Institutional Review Board (IRB # 48678). IECs from the duodenum samples were cultured as described previously [25, 26]. Briefly, samples from the operating room were immediately placed in ice-cold PBS. The intestinal fragments were incubated in ice-cold PBS containing 10 mM EDTA for 60 min at 4 °C. After incubation, crypts were released by shaking with ice-cold PBS. Crypt suspensions were collected and passed through a 100 μm cell strainer and centrifuged at 300 × g for 5 min. Isolated crypts were mixed with Matrigel (Corning) and cultured in IntestiCult™ Organoid Growth Medium (Stemcell Technologies, #100-0340). To determine the role of miR-181a-5p, human duodenum organoids were incubated with either miR-181a-5p miRCURY LNA miRNA inhibitor or miRNA negative control oligos (Qiagen, Germantown, MD). Human duodenum organoids were infected with lentivirus expressing miR-181a-5p mimic (HMI0268, Sigma) or miRNA mimic control (NCLMIR002, Sigma). The infected organoids were cultured in the presence of puromycin (2 μg/ml) to select for organoid cells overexpressing of miR-181a-5p.

### Western blot analysis

Western blotting was performed as we have previously described [27]. Total protein was resolved on a 10% polyacrylamide gel and transferred to polyvinylidene fluoride membranes. Membranes were incubated for 1 h at room temperature in blotting solution and then incubated in primary antibody diluted in 5% bovine serum albumin solution containing 0.02% sodium azide overnight at 4 °C. Antibodies to Cyclin D1 (ab134175) and KRT20 (ab854) from Abcam (Cambridge, UK), OLFM4 (#39141), APOA4 (#5700), FABP1 (#13368), HK1 (#2024), Na-K ATPase (#3010) (all from Cell Signaling, Danvers, MA) and HK2 (GeneTex, Irvine, CA; GTX124375), VILLIN (sc-7672, Santa Cruz Biotechnology, Dallas, TX), and β-actin (A1978, Sigma) were used, and following blotting with a horseradish peroxidase-conjugated secondary antibody, protein expression was visualized using an enhanced chemiluminescence (ECL) detection system.

### Quantitative real time RT-PCR (qPCR) analysis

Total RNA was extracted using a RNeasy Mini Kit (Qiagen) and treated with DNase (RQ1, Promega, Madison, WI). Synthesis of cDNA was performed using reagents in the TaqMan Reverse Transcription Reagents Kit (Applied Biosystems, Foster City, CA). TaqMan probe and primers for human and mouse KRT20, intestinal alkaline phosphatase (IAP), APOA4, FABP1, VILLIN and GAPDH were purchased from Thermo Fisher Scientific (Waltham, MA). All values were normalized to GAPDH levels. To determine the expression of mature miRNA species, total RNA was extracted using a mirVana miRNA Isolation Kit (Thermo Fisher Scientific). cDNA for miRNA gene expression analysis was synthesized using a TaqMan™ Advanced miRNA cDNA Synthesis Kit (Thermo Fisher Scientific). The following TaqMan assays were used: hsa-miR181a-5p Assay ID: 477857_mir, mm-miR181a-5p Assay ID: mmu481485_mir, mm-miR-181b-5p Assay ID: mmu478583_mir, mm-miR-181c-3p Assay ID: mmu481437_mir, mm-miR-181d-5p Assay ID: mmu479517_mir, and U6 snRNA Assay ID: 001973 (Thermo Fisher Scientific). For mature miRNA expression levels, all values were normalized to U6 levels. qPCR analysis was performed with using QuantStudio Real-Time PCR System (Thermo Fisher Scientific). [19, 20].

### Seahorse analysis

Intestinal crypts were isolated and seeded in 24-well plates and treated with either control oligos or miR-181a-5p inhibitor or PBS control or BMP4. The organoids were transferred to XFe96 cell culture microplates before measurement. Extracellular acidification rate (ECAR) and oxygen consumption rate (OCR) was measured using the XFe96 analyzer (Agilent Technologies, Santa Clara, CA) according to the manufacturer’s instructions as described [19, 28]. Each plotted value was normalized to total amount of DNA present in all wells of the various groups.

### IF, Immunohistochemistry (IHC) and IAP staining

Organoids grown in Matrigel were harvested in cold PBS followed by centrifugation at 300 × g for 5 min. Organoids were fixed with 4% formaldehyde at room temperature for 1 h, centrifuged at 300 × g for 5 min to remove the 4% formaldehyde, and resuspended in HISTOGEL (Thermo Scientific; HG-4000-012). The HISTOGEL containing organoids were allowed to cool to room temperature and stored in 70% ethanol at 4 °C until ready for paraffin embedding. Paraffin-embedded organoid sections were processed for routine IF or IHC staining. DAPI (Electron Microscopy Sciences, Hatfield, PA; 17989-20) was used as a counterstain to detect nuclei. Images were acquired with a confocal microscope (Nikon ECLIPSE Ti2) at 40× magnification. IHC staining was performed as we have described previously [29]. Tissues were processed for routine IF or IHC staining using the following antibodies: anti-KRT20 (Abcam; ab854), anti-Na,K-ATPase (Cell Signaling; #3010), anti-VILLIN (Santa Cruz Biotechnology; sc-58897) and anti-Ki67 (R&D Systems, Minneapolis, MN; MAB7617), E-cadherin (Cell Signaling; #3195) and cyclin D1 (Abcam; ab134175). Negative controls (including no primary antibody or isotype-matched mouse immunoglobulin G) were used in each assessment. Image analysis was performed using ImageJ. Image contrast was adjusted for visualization purposes, and quantification was always applied on raw, non-adjusted images. Image density per organoid was quantified and normalized to the total cell number per organoid.

Mouse SI tissues were harvested and fixed with 4% formaldehyde overnight. Paraffin-embedded mouse SI sections injected with control oligos or miR-181a-5p inhibitor were processed for routine IHC staining for cyclin D1 (Abcam; ab134175); anti-Ki67 (R&D Systems, Minneapolis, MN; MAB7617) as we have described previously [19, 20]. IAP staining was performed using Vulcan Fast Red Chromogen kit (Biocare Medical, Concord, CA), following the manufacturer’s recommendations as we have described [20].

### In vitro IAP activity assay

Organoids grown in Matrigel were harvested in cold PBS followed by centrifugation. Organoid cell lysates were used to determine IAP activity by a commercially available kit (AP0100, Sigma) as we have described [30, 31].

### miRNA-seq and analysis

RNA was isolated using a mirVana miRNA Isolation Kit (Thermo Fisher Scientific). miRNA-seq was performed by the Genomics, Epigenomics and Sequencing Core (GES Core) at the University of Cincinnati. To construct the library, NEBNext Small RNA Sample Library Preparation kit (NEB, Ipswich, MA) was used with a modified approach for precise library size selection with high sensitivity. Briefly, after 15 cycles of final PCR, the libraries were mixed with custom-designed DNA ladder that contains 135 and 146 bp DNA. This size range corresponds to miRNA library with 16–27 nt insert that covers all miRNAs. After high resolution agarose gel electrophoresis, the library pool ranging from 135–146 bp, including the DNA marker, was gel purified and quantified by NEBNext Library Quant kit (NEB) using QuantStudio 5 Real-Time PCR System (Thermo Fisher Scientific). The first sequencing was performed using a NextSeq 2000 sequencer (Illumina, San Diego, CA) to generate a few million (M) reads to quantify the relative concentration of each library. The volume of each library was then adjusted to generate >3 M reads per sample in the second sequencing for final data analysis.

The adaptors and low-quality sequences were trimmed from the miRNA-seq reads using bbmap (v38.73); reads with lengths <15 nucleotides and longer than 27 nucleotides were discarded. For quantification of mature miRNAs documented in miRBase [32], sequencing reads were first mapped to the reference genome using the mapper.pl script in the miRDeep2 (v0.1.3) package [33]. The quantifier.pl script was used with default settings to quantify the number of reads mapped to mature miRNAs. The R package EdgeR [34] was used to identify differentially expressed genes among different experimental contrasts.

### Statistical analysis

Pairwise comparisons between two groups were performed using two-sample *t*-test or analysis of variance for multiple groups with contrast statements. Linear mixed models were used for mice in vivo studies comparing control oligos and miR-181a-5p inhibitor to account for multiple observations from multiple crypts and villi per mouse. Adjustment in *p*-values due to multiple pairwise testing between groups was performed using the Holm’s step-down procedure. Three replicates were used for each cell culture condition and each experiment was repeated at least three times. Model fit and statistical test assumptions were assessed and appropriate data transformation (log) was employed as indicated. Mice within a cage were randomized to both groups in the experiment to ensure balance in treatment group assignments across all cages. The animals were randomly selected for group assignment without preference to size or other confounding factors. Furthermore, only animal IDs without information on group assignment were available to staff performing the endpoint evaluation. All data from animal samples with measurement of study endpoints were included in the analysis. For the mice in vivo studies, sufficient sample size was utilized to provide at least 80% power to detect large effect sizes (at least 2.0 mean differences in S.D. units) based on a two-group comparison, two-sided test with 5% significance level. Bar graphs represent mean ± SD levels in each group; *p*-values < 0.05 were considered statistically significant.

## Results

### BMP4 induces enterocyte differentiation and inhibits stem cell activity in human duodenum and mouse SI organoids

**Fig. 1:**
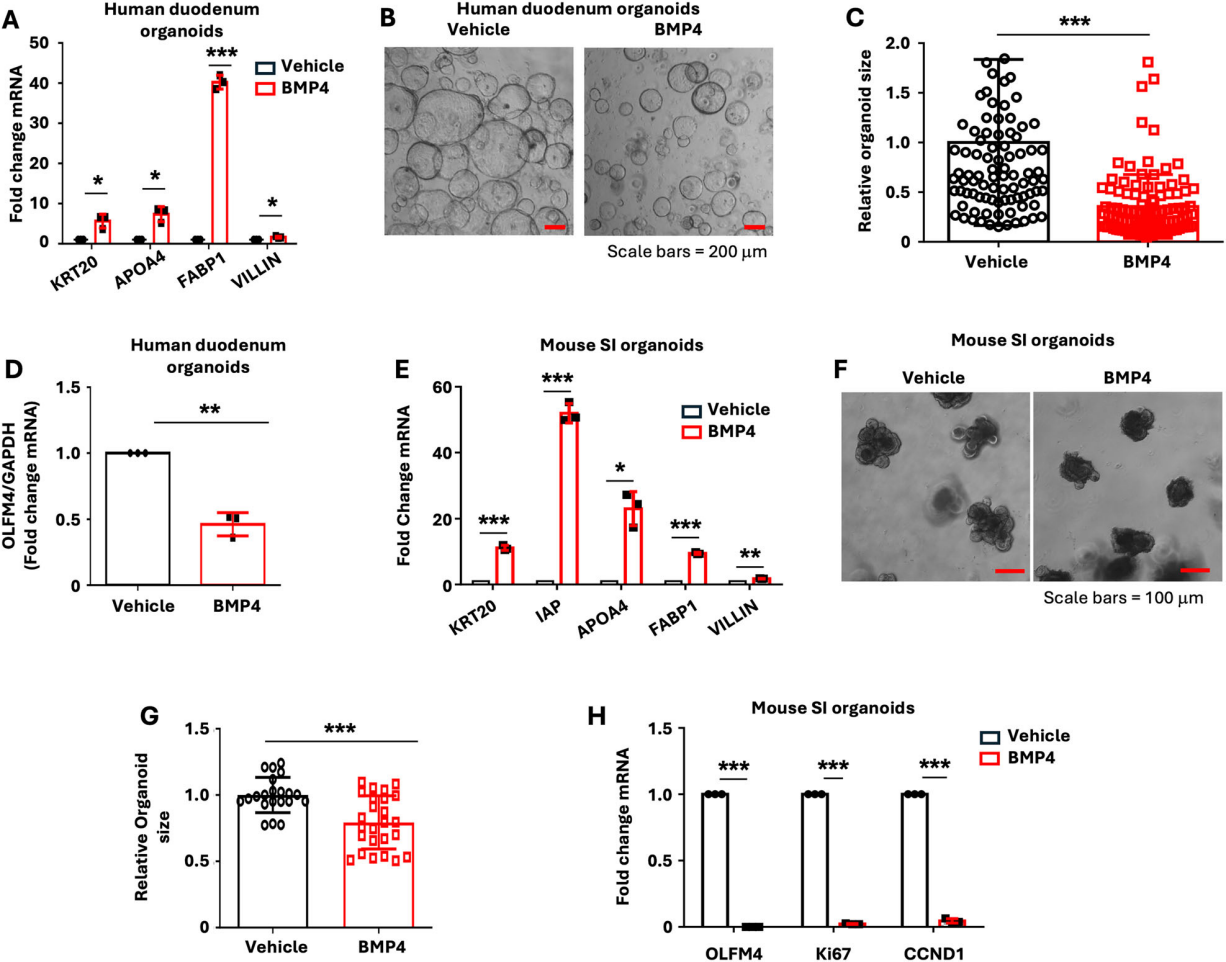
BMP4 increased enterocyte marker expression and inhibited intestinal stemness in human duodenum and mouse SI organoids. **A**–**D** Human duodenum organoids were cultured in the presence of BMP4 (500 ng/ml) or control PBS for 4 d. Total RNA was extracted, and KRT20, APOA4, FABP1, and VILLIN mRNA expression was assessed by qPCR (**A**). Data are normalized to GAPDH; *n* = 3 biological repeats. Representative image of organoids from control PBS and BMP4 treatment groups is shown in B with quantification of the organoid area shown in (**C**) (98 organoids per PBS group and 105 organoids per BMP4 group were measured). qPCR analysis of OLFM4 expression (**D**). **E**–**H** Mouse SI organoids were cultured in the presence of BMP4 (500 ng/ml) or control PBS for 4 d. qPCR analysis of selected enterocyte markers in mouse SI organoids differentiated in the absence or presence of BMP4 (**E**). Data are normalized to GAPDH; *n* = 3 biological repeats. Representative image of organoids from control PBS and BMP4 treatment is shown in (**F**). Quantification of the organoid area is shown in (**G**) (21 organoids per PBS group and 25 organoids per BMP4 group were measured). OLFM4, Ki67, and CCND1 mRNA expression were assessed by qPCR (**H**). Data are normalized to GAPDH; *n* = 3 biological repeats. ^∗^*p* < 0.05; ^∗∗^*p* < 0.01; ^∗∗∗^*p* < 0.005.

To better delineate the roles of BMP4 in human IECs, we first used human small intestinal organoids. These organoid cultures demonstrate ISC self-renewal and multipotential differentiation under certain in vitro conditions [25, 26]. Treatment of the organoids with recombinant human BMP4 protein for 4 d increased the expression of KRT20, APOA4, FABP1, and VILLIN (Fig. 1A), markers of mature enterocytes, demonstrating a contributary role of BMP4 to enterocyte differentiation in human organoids.

We next determined the effects of BMP4 on growth of human duodenum organoids. The activity of ISCs was assessed based on their ability to drive the formation of organoids [20, 35]. Treatment of human duodenum organoids with BMP4 led to the formation of smaller organoids (Fig. 1B, C). Consistent with these findings, mRNA levels of OLFM4, an ISC marker, were decreased in BMP4-treated organoids as compared with control (Fig. 1D). Our results demonstrated a role for BMP4 in repression of human ISC self-renewal (i.e., lower expression of the ISC marker OLFM4 with small organoids). Similarly, treatment of mouse SI organoids with BMP4 increased enterocyte differentiation as noted by the increased expression of the enterocyte markers KRT20, IAP, APOA4, FABP1, and VILLIN (Fig. 1E), and inhibited IEC proliferation as noted by decreased organoid size (Fig. 1F–G) and expression of OLFM4 and proliferating cell markers Ki67 and CCND1 (Fig. 1H). Together, these findings demonstrate a critical role for BMP4 in human and mouse enterocyte maturation and IEC proliferation.

### miR-181 family members are downstream targets of BMP4

miRNAs play important roles in controlling the development of fetal tissues [36], whereas miRNAs that are responsible for promoting IEC differentiation remain poorly understood.

**Fig. 2:**
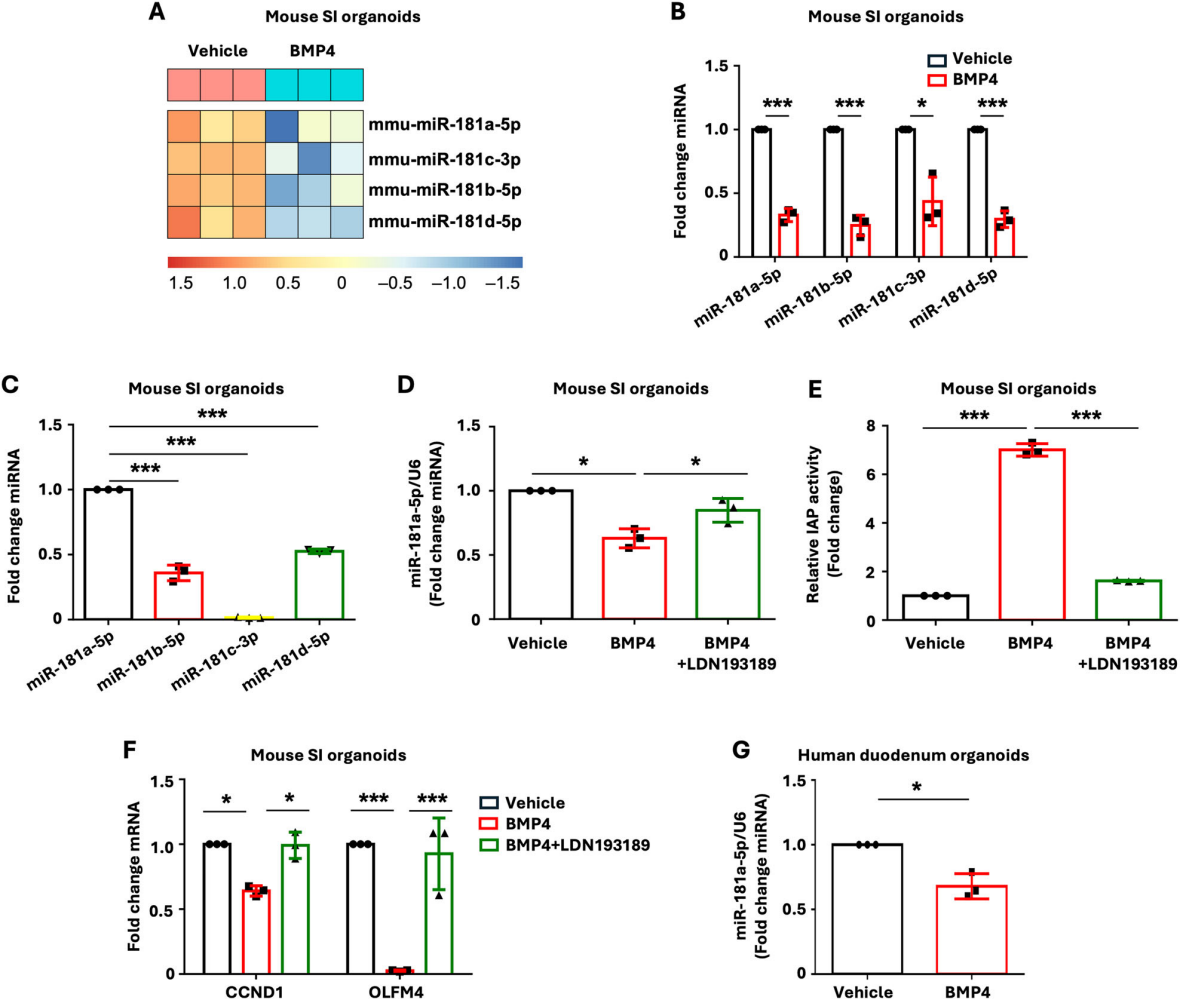
miR-181 family is a downstream target of BMP4 in IECs. **A**–**C** Mouse SI organoids were treated with or without BMP4 (500 ng/ml) for 4 d. Bulk miRNA sequencing was performed. **A** Heatmaps depict the miR-181 family members significantly downregulated upon BMP4 stimulation. Heatmaps display row *Z* scores of CPM (counts per million) values. The BMP4-mediated inhibition of miR-181 expression was validated by qPCR (**B**). Relative expression of miR-181 members showed highest expression of miR-181a-5p in mouse SI organoids (**C**). **D**–**F** Inhibition of BMP signaling attenuated BMP4-induced repression of miR-181a-5p expression and enterocyte differentiation. Mouse SI organoids were treated with or without BMP4 (500 ng/ml) in the presence or absence of BMP signaling inhibitor LDN-193189 (1 μM) for 4 d. Expression of miR-181a-5p was determined by qPCR (**D**). IAP activity was determined (**E**). ISC proliferation markers were determined by qPCR (**F**). **G** Human duodenum organoids were treated with or without BMP4 (500 ng/ml) for 4 d. Expression of miR-181a-5p was determined by qPCR; *n* = 3 biological repeats. ^∗^*p* < 0.05; ^∗∗∗^*p* < 0.005.

To characterize the miRNA profile associated with BMP4-induced IEC differentiation, mouse SI organoids were treated with BMP4, and global small RNA sequencing was performed. miRNA analysis revealed differentially expressed miRNAs in response to BMP4-induced differentiation (data deposited in Gene Expression Omnibus as GSE266339). Among the altered miRNAs, four miR181 family members, including miR-181a-5p, b-5p, c-3p, and d-5p, were significantly downregulated in BMP4-treated IECs (Fig. 2A). The decreased expression of miR-181 family members was further determined and validated by qPCR (Fig. 2B). To further dissect the role of BMP4 signaling, we used a selective chemical inhibitor for BMP type I receptor kinases, LDN-193189 [37, 38]. As miR-181a-5p is the most highly expressed member of the miR-181 family in IECs (Fig. 2C), we further determined the regulation of miR-181a-5p by BMP4 signaling. Treatment of mouse SI organoids with BMP4 decreased miR-181a-5p expression; this decrease was attenuated by combination treatment with LDN-193189 (Fig. 2D). In addition, treatment with LDN-193189 attenuated the BMP4-induced increase in IAP activity, an enterocyte marker, (Fig. 2E) and increased IEC proliferation repressed by BMP4 (Fig. 2F). Similarly, the BMP4-repressed expression of miR-181a-5p in human duodenum organoids was also demonstrated (Fig. 2G). These findings suggest that miR-181a-5p plays a critical role for BMP4 signaling in the induction of IEC differentiation and repression of IEC proliferation.

### miR-181a-5p regulates mouse IEC differentiation and proliferation

**Fig. 3:**
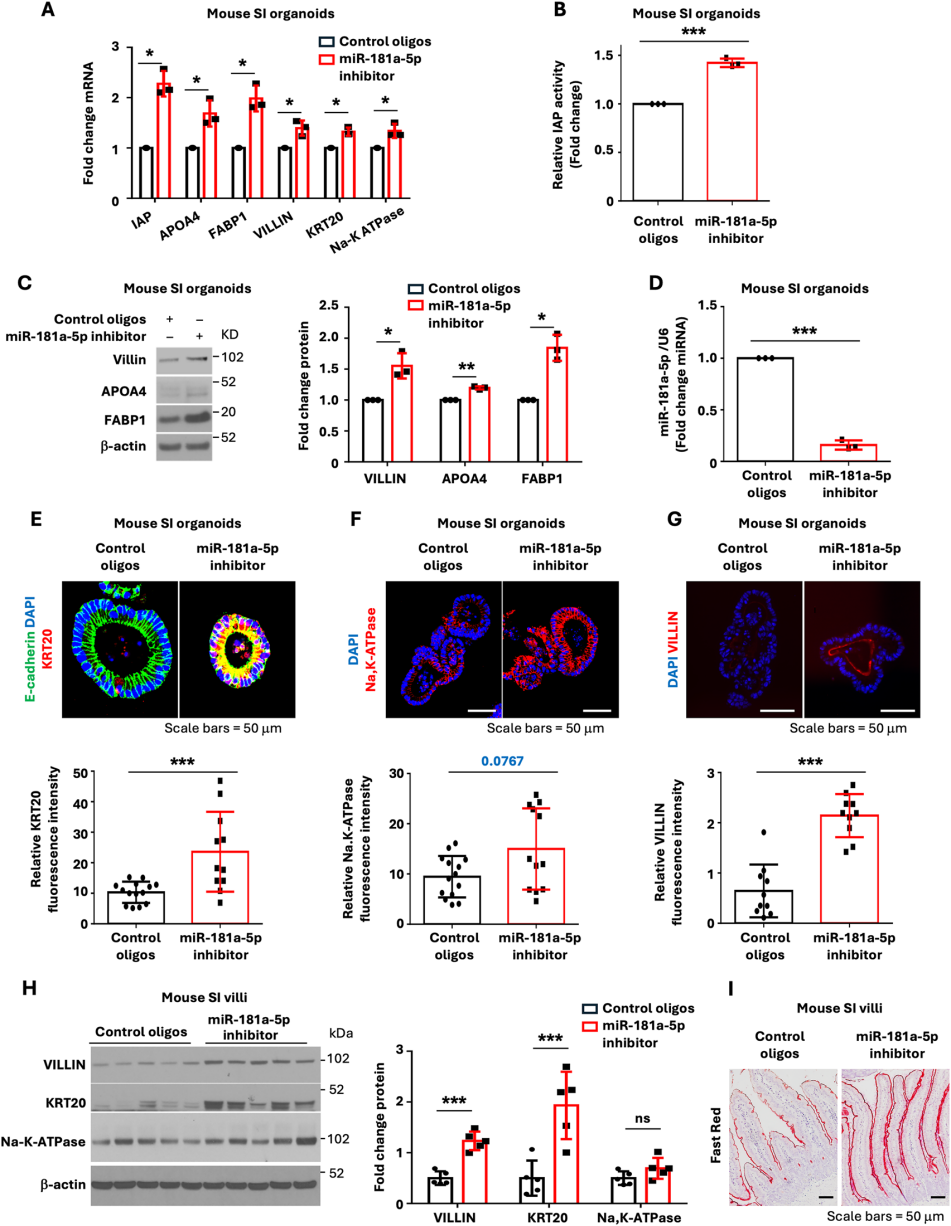
Inhibition of miR-181a-5p increased enterocyte marker expression in mouse SI organoids. Mouse SI organoids were cultured in the presence of LNA miR-181a-5p inhibitor or control oligos (0.75 μM) for 5 d. **A** qPCR analysis of selected enterocyte markers; *n* = 3 biological repeats. **B** Mouse organoid lysates were extracted, and IAP activity determined; *n* = 3 biological repeats. **C** Treatment with LNA miR-181a-5p inhibitor increased enterocyte marker protein expression. The images are representative of three independent experiments. VILLIN, APOA4, and FABP1 signals from three separate experiments were quantitated by densitometer and expressed as fold change with respect to *β*-actin. **D** LNA miR-181a-5p inhibitor decreased endogenous miR-181a-5p expression; *n* = 3 biological repeats. **E**–**G** Microscopy analysis of enterocytes in mouse SI organoids. The relative fluorescence intensities of KRT20 (control oligos *n* = 14 organoids; miR-181a-5p inhibitor *n* = 11 organoids), Na,K-ATPase (control oligos *n* = 14 organoids; miR-181a-5p inhibitor *n* = 12 organoids), and VILLIN (control oligos *n* = 10 organoids; miR-181a-5p inhibitor *n* = 10 organoids) staining were quantified using ImageJ. **H**–**J** C57BL/6 mice were injected IP with control oligos (*n* = 5 mice) or miR-181a-5p inhibitor (*n* = 5 mice). Seven d after injection, mouse SI were harvested and analyzed. Western blot analysis (**H**). Image signals from five mice were quantitated by densitometer and expressed as fold change with respect to *β*-actin. Representative Fast Red staining of the small intestine revealed an increase in IAP activity (**J**). ^∗^*p* < 0.05; ^∗∗^*p* < 0.01; ^∗∗∗^*p* < 0.005.

We have shown that treatment with BMP4 significantly repressed miR-181 expression. Since miR-181a-5p is the most highly expressed member of the miR-181 family (Fig. 2C), we next determined the role of miR-181a-5p in IECs. Mouse SI organoids were infected with lentivirus expressing miR-181a-5p mimic or miRNA mimic control as described [22] followed by RNA-seq analysis. RNA analysis revealed differentially expressed RNAs in response to overexpression of miR-181a-5p (data deposited in Gene Expression Omnibus as GSE294782). Importantly, overexpression of miR-181a-5p decreased enterocyte marker expression (Supplementary Table 1). Consistent with the results from RNA-seq analysis, overexpression of miR-181a-5p mimic repressed the expression of enterocyte markers IAP and FABP1 determined by qPCR (Supplementary Fig. 1). To further determine the role of miR-181a-5p in the intestine, mouse SI organoids were incubated with an LNA miR-181a-5p inhibitor, and differentiation was assessed. LNA miRNA inhibitors are antisense oligonucleotides and can be added directly to culture medium without the need for transfection reagents, thus providing efficient miRNA inhibition [39]. In line with the inhibition of enterocyte differentiation by miR-181a-5p mimic, the LNA miR-181a-5p inhibitor increased enterocyte differentiation as noted by increased mRNA expression of the enterocyte markers IAP, APOA4, FABP1, VILLIN, KRT20, and Na,K-ATPase (Fig. 3A), IAP activity (Fig. 3B), and protein expression of the enterocyte markers VILLIN, APOA4, and FABP1 (Fig. 3C), associated with the repression of endogenous miR-181a-5p expression (Fig. 3D). Inhibition of enterocyte differentiation by miR-181a-5p was further evidenced by IF analysis of KRT20 (Fig. 3E), Na,K-ATPase (Fig. 3F), and VILLIN (Fig. 3G).

As further confirmation of the effect of miR-181a-5p on intestinal cell differentiation and proliferation in vivo, we treated mice with the miR-181a-5p inhibitor. miRCURY LNA miRNA inhibitors can efficiently inhibit target miRNA activity in vivo directly by IP injection [24]. C57BL/6 mice (8-10 wks old) were injected with miR-181a-5p miRCURY LNA miRNA inhibitor (20 mg/kg) or miRCURY LNA control oligos (20 mg/kg) as described [24]. Administration of miR-181a-5p inhibitor repressed miR-181a-5p levels in mouse epithelial cells in SI and colon (Supplementary Fig. 2). Importantly, miR-181a-5p inhibitor increased intestinal expression of VILLIN, KRT20, and Na,K-ATPase protein (Fig. 3H) and increased IAP activity as noted by Fast Red staining (Fig. 3I), demonstrating that miR-181a-5p inhibits intestinal enterocyte differentiation.

**Fig. 4:**
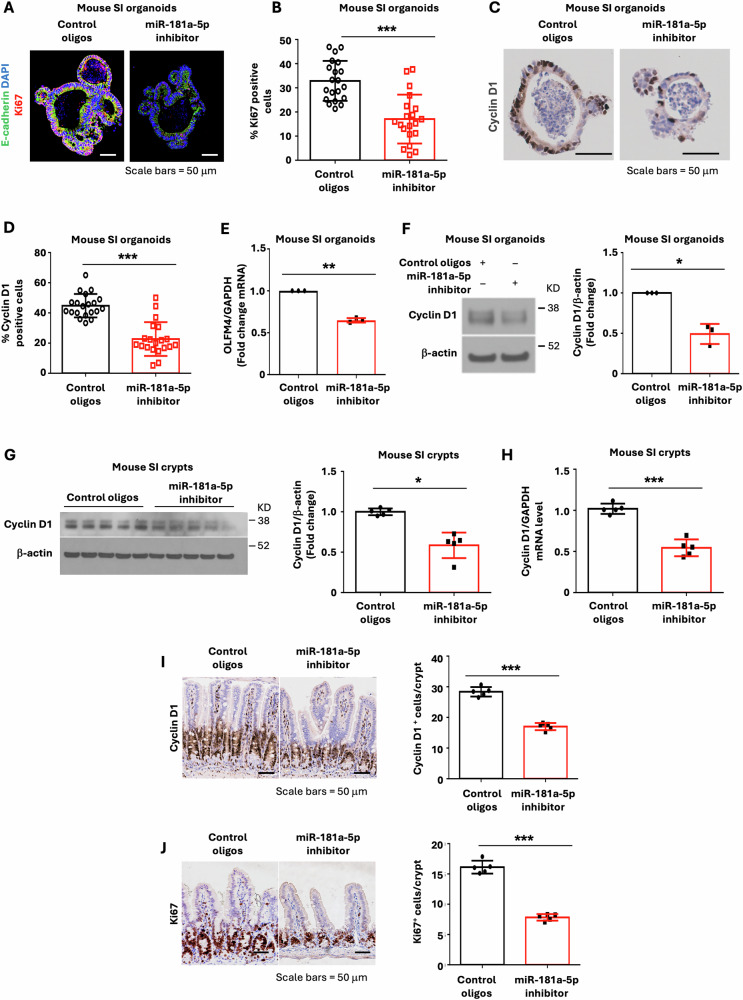
miR-181a-5p inhibitor decreased stemness in mouse SI organoids. Mouse SI organoids were cultured in the presence of LNA miR-181a-5p inhibitor or control oligos (0.75 μM) for 5 d. **A**, **B** Organoids were stained with anti-Ki67 antibody to detect proliferating cells and visualized as shown in (**A**). The percentage of Ki67 positive cells was quantified comparing organoids treated with LNA miR-181a-5p inhibitor or control oligos (**B**) (*n* = 20). **C**, **D** IHC staining for proliferation marker cyclin D1. Cyclin D1 positive cells were visualized as shown in (**C**). The percentage of cyclin D1 positive cells was quantified (**D**) (*n* = 20). **E** qPCR analysis of stem cell marker OLFM4 mRNA expression; *n* = 3 biological repeats. **F** Western blot analysis of cyclin D1 protein expression. The images are representative of three independent experiments. Cyclin D1 signals from three separate experiments were quantitated by densitometer and expressed as fold change with respect to *β*-actin. **G–J** C57BL/6 mice were injected IP with control oligos or miR-181a-5p inhibitor. Seven d after injection, mouse SI was harvested and analyzed. Western blot analysis (**H**). Cyclin D1 signals from five mice were quantitated by densitometer and expressed as fold change with respect to *β*-actin. Cyclin D1 mRNA levels were determined by qPCR (**H**). IHC staining for cyclin D1 (**I**) and Ki67 (**J**) demonstrated that the number of positive cells was decreased after in SI treated with miR-181a-5p inhibitor. (*n* = 5 mice, 20 crypts per mouse). ^∗^*p* < 0.05; ^∗∗^*p* < 0.01; ^∗∗∗^*p* < 0.005.

Since BMP4 has shown an inhibitory role in ISC renewal, we next determined the effects of miR-181a-5p inhibition on IEC proliferation. As shown in Fig. 4, treatment with LNA miR-181a-5p inhibitor inhibited IEC proliferation as noted by the decreased numbers of Ki67 (Fig. 4A, B) and cyclin D1 (Fig. 4C, D) positive cells and expression of OLMF4 mRNA (Fig. 4E) and cyclin D1 protein (Fig. 4F). These findings demonstrate that miR-181a-5p contributes to the regulation of mouse enterocyte differentiation and IEC proliferation. Along with the increased differentiation, the expression of cyclin D1 (Fig. 4G, H) and the positive cell number of cyclin D1 (Fig. 4I) and Ki67 (Fig. 4J) were decreased in intestinal epithelial cells from mice treated with miR-181a-5p inhibitor, demonstrating the decreased proliferation. Therefore, consistent with the results from ex vivo studies, the results from our in vivo studies further demonstrate that miR-181a-5p is critical for the regulation of intestinal cell proliferation and differentiation.

### miR-181a-5p regulates human IEC differentiation and stemness

**Fig. 5:**
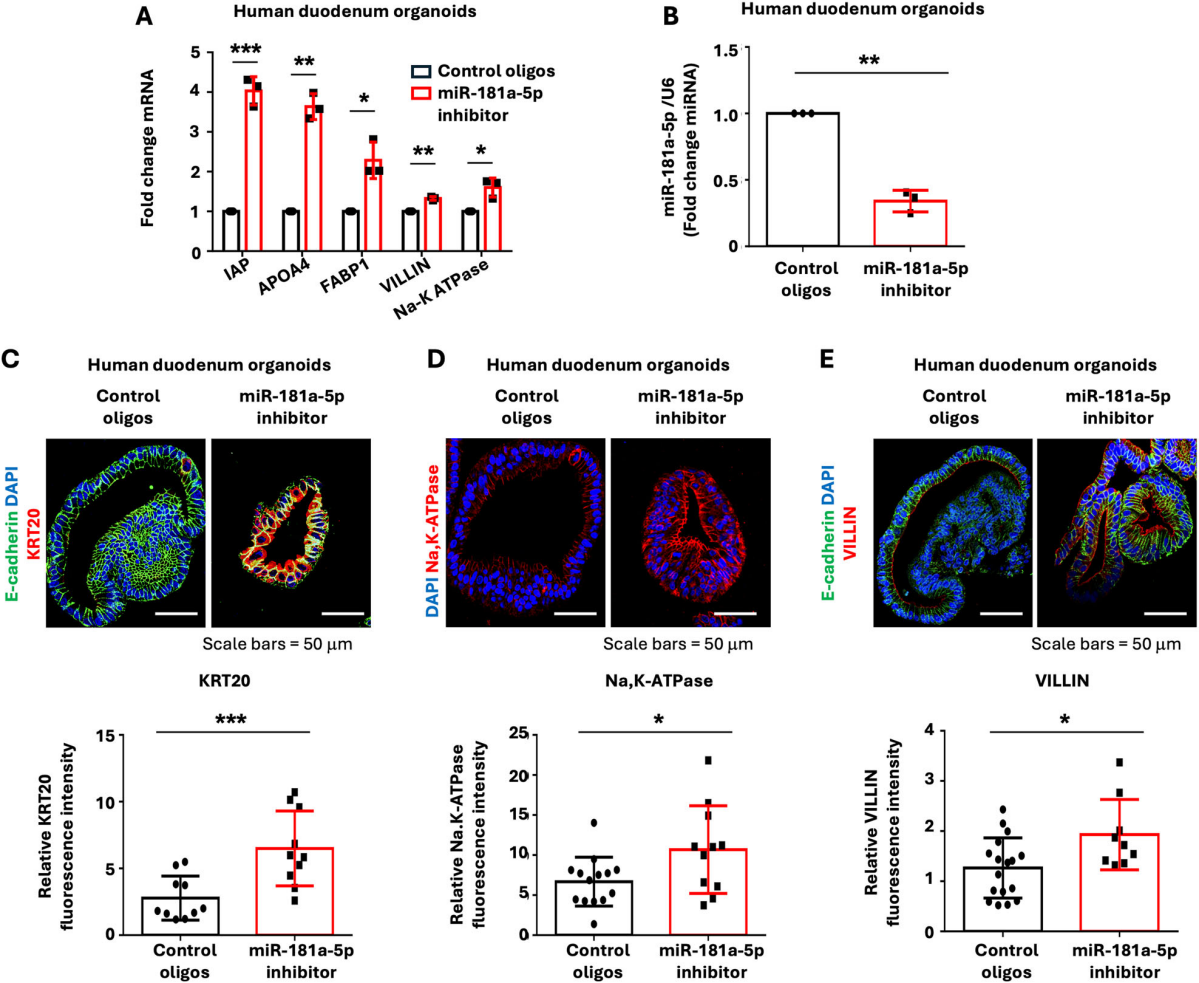
Inhibition of miR-181a-5p increased enterocyte marker expression in human duodenum organoids. Human duodenum organoids were cultured in the presence of LNA miR-181a-5p inhibitor or control oligos (1 μM) for 7 d. **A** qPCR analysis of enterocyte markers IAP, APOA4, FABP1, VILLIN, and Na,K-ATPase expression; *n* = 3 biological repeats. **B** Treatment with LNA miR-181a-5p inhibitor decreased endogenous miR-181a-5p expression; *n* = 3 biological repeats. **C**–**E** IF staining of organoids for KRT20 (**C**), Na,K-ATPase (**D**), and VILLIN (**E**). The relative fluorescence intensity of KRT20 (control oligos *n* = 10 organoids; miR-181a-5p inhibitor *n* = 10 organoids), Na,K-ATPase (control oligos *n* = 14 organoids; miR-181a-5p inhibitor *n* = 11 organoids), and VILLIN (control oligos *n* = 17 organoids; miR-181a-5p inhibitor *n* = 9 organoids) staining was quantified using ImageJ fluorescence analyzer. ^∗^*p* < 0.05; ^∗∗^*p* < 0.01; ^∗∗∗^*p* < 0.005.

**Fig. 6:**
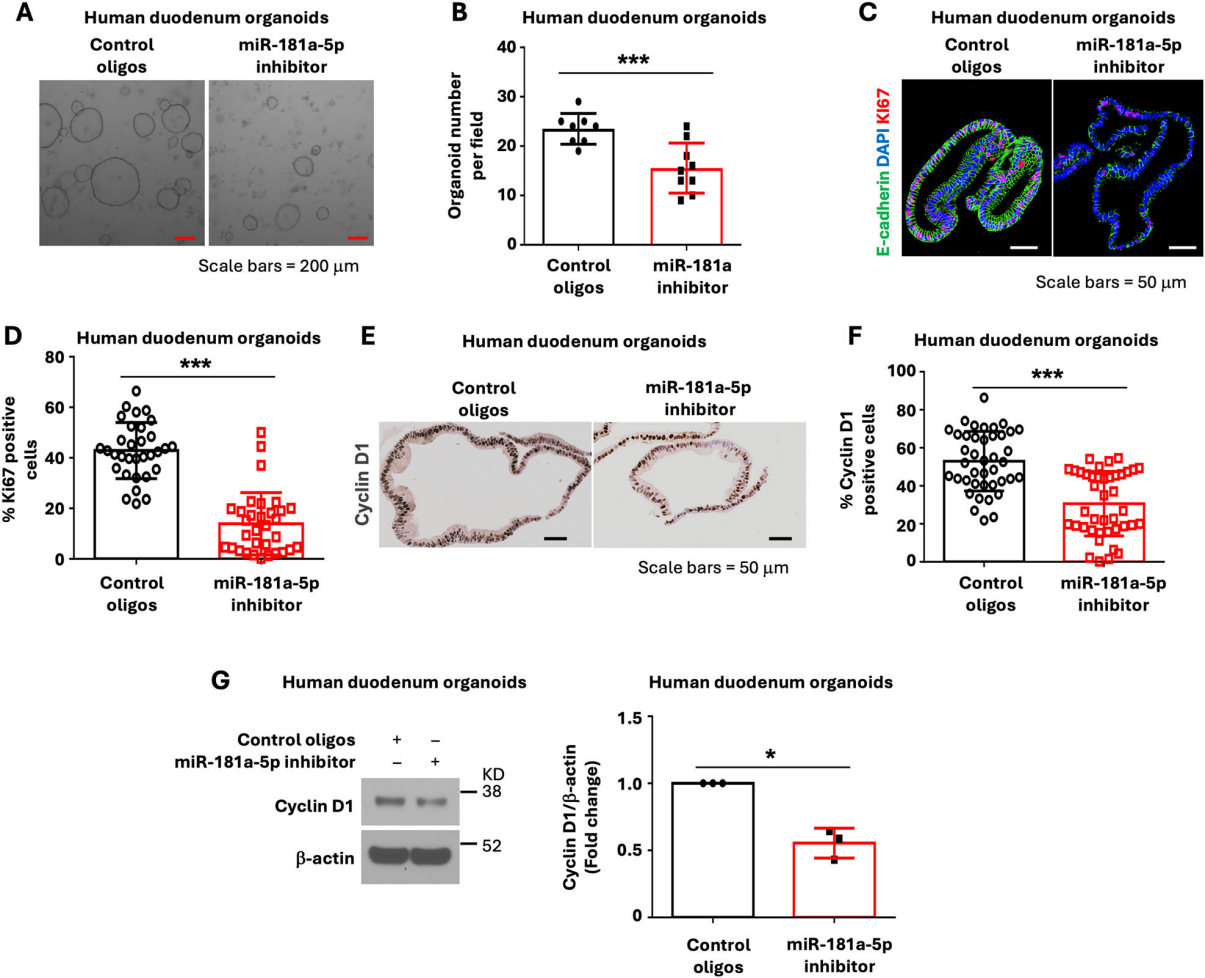
Repression of miR-181a-5p resulted in the inhibition of human IEC proliferation. Human duodenum organoids were cultured in the presence of LNA miR-181a-5p inhibitor or control oligos (1 μM) for 7 d. **A**, **B** Organoid morphology was assessed by microscopy as shown in (**A**) Organoid-forming assay is shown in **B** (*n* = 8 ~ 9 fields per group). **C**, **D** IF staining for Ki67 positive cells as an assessment of cell proliferation (**C**). The percentage of Ki67 positive cells was quantified (**D**) (*n* = 20). **E**, **F** IHC staining for proliferation marker cyclin D1 is shown in (**E**). The percentage of cyclin D1 positive cells were quantified (**F**). (*n* = 20). **G** Western blot analysis of cyclin D1 protein expression; images are representative of three independent experiments. Cyclin D1 signals from three separate experiments were quantitated by densitometer and expressed as fold change with respect to *β*-actin. ^∗^*p* < 0.05; ^∗∗∗^*p* < 0.005.

To determine whether miR-181a-5p plays a similar role in human IECs, human duodenum organoids were incubated with the LNA miR-181a-5p inhibitor or control oligos and differentiation was assessed. As shown in Fig. 5, the LNA miR-181a-5p inhibitor increased enterocyte differentiation as noted by increased expression of the enterocyte markers IAP, APOA4, FABP1, VILLIN, and Na,K-ATPase (Fig. 5A). The repression of endogenous miR-181a-5p by LNA miR-181a-5p inhibitor was also confirmed (Fig. 5B). Increased protein expression of the enterocyte markers KRT20 (Fig. 5C), Na,K-ATPase (Fig. 5D), and VILLIN (Fig. 5E) was further demonstrated by IF staining. In contrast, overexpression of miR-181a-5p repressed the basal and BMP4-induced expression of enterocyte differentiation markers in human duodenum organoids (Supplementary Fig. 3-4). Moreover, the LNA miR-181a-5p inhibitor repressed human IEC stemness as noted by decreased organoid numbers (Fig. 6A, B), decreased numbers of Ki67 (Fig. 6C, D) and cyclin D1 positive cells (Fig. 6E, F), and cyclin D1 protein expression (Fig. 6G). Together, our results demonstrate a role for miR-181a-5p in the regulation of proliferation and differentiation in mouse and human IECs. Importantly, our results suggest a predominant role for miR-181a-5p, acting downstream of BMP4, in the maintenance of intestinal homeostasis.

### BMP4/miR-181a-5p pathway regulates glycolysis in IECs

**Fig. 7:**
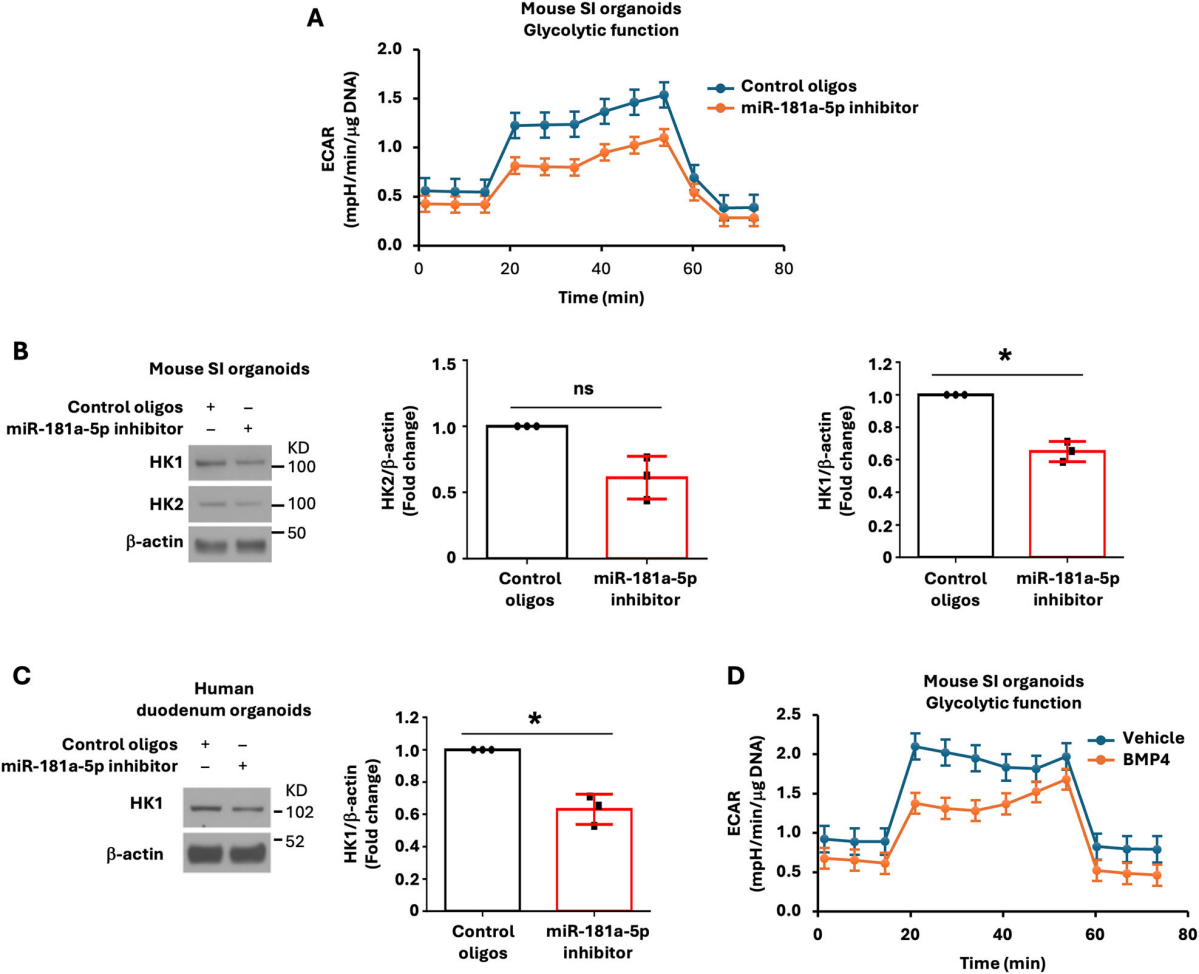
BMP4/miR-181a-5p regulates glycolysis in IECs. **A** Mouse SI organoids were treated with LNA miR-181a-5p inhibitor or control oligos (0.75 μM) for 4 d followed by Seahorse analysis of metabolism (*n* = 4 biological repeats). **B** Mouse SI organoids were treated with LNA miR-181a-5p inhibitor or control oligos (0.75 μM) for 5 d. Cells were lysed and western blot analysis was performed using antibodies against HK1, HK2, and *β*-actin. The images are representative of three independent experiments. HK1 and HK2 signals from three separate experiments were quantitated by densitometer and expressed as fold change with respect to *β*-actin. **C** Human duodenum organoids were treated with LNA miR-181a-5p inhibitor or control oligos (1 μM) for 7 d. Cells were lysed and western blot analysis was performed using antibodies against HK1 and *β*-actin. The images are representative of three independent experiments. HK1 signals from three separate experiments were quantitated by densitometer and expressed as fold change with respect to *β*-actin. **D** Mouse SI organoids were treated with BMP4 (0.5 μg/ml) for 4 d and metabolism assessed by Seahorse (*n* = 4 biological repeats). ^∗^*p* < 0.05.

We and others have shown an essential role for glycolysis in the maintenance of ISC function [19, 21]. We next determined the effects of BMP4/miR-181a-5p on the metabolism of mouse intestinal organoids using Seahorse Extracellular Flux analysis. Treatment with the miR-181a-5p inhibitor significantly repressed ECAR (Fig. 7A), which is associated with decreased glycolytic capacity. In contrast, treatment with the miR-181a-5p inhibitor had only a minor effect on OCR (Supplementary Fig. 5). To determine how miR-181a-5p affects glycolysis, the expression of glycolytic enzymes involved in glycolysis was determined. Treatment with the miR-181a-5p inhibitor decreased HK1 protein expression in mouse IECs (Fig. 7B) and human IECs (Fig. 7C), suggesting that miR-181a-5p regulation of glycolysis is through regulation of HK1 expression. Consistently, treatment with BMP4 significantly repressed ECAR (Fig. 7E) in mouse IECs. Therefore, these results suggest that BMP4 inhibits glycolysis through repression of miR-181a-5p expression.

### HK1-dependent glycolysis regulates enterocyte differentiation

**Fig. 8:**
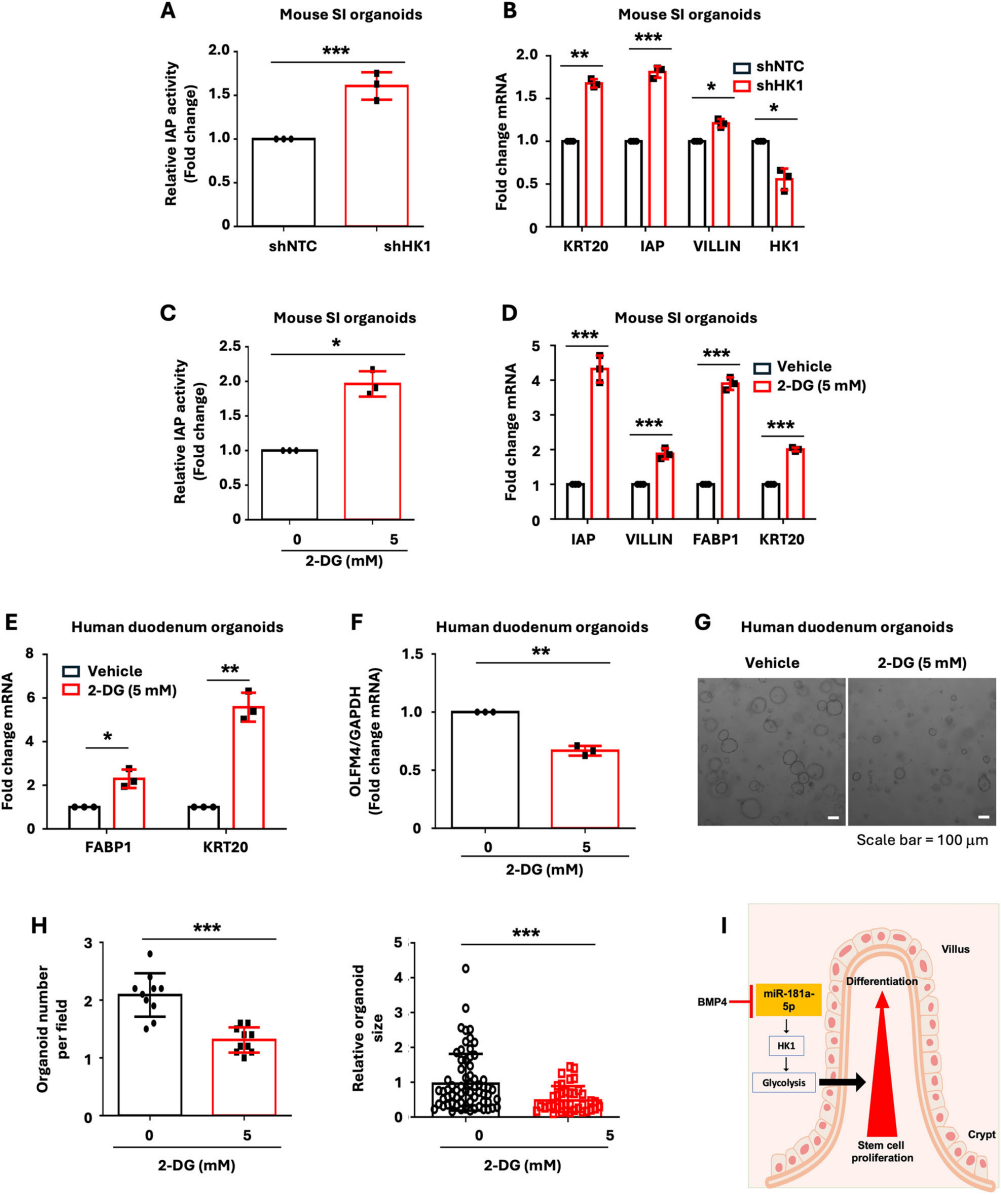
HK1-dependent glycolysis regulates intestine enterocyte differentiation. **A**, **B** Mouse SI organoids were infected with lentivirus expressing the HK1 shRNA or NTC shRNA and cultured in the presence of puromycin (2 μg/ml) to select for organoid cells with stable knockdown of HK1. The organoids with stable knockdown of HK1 were incubated for 5 d followed by extraction of cell lysates to measure IAP activity as an indication of differentiation (**A**). Total RNA was extracted, and qPCR performed; *n* = 3 biological repeats (**B**). **C**, **D** Mouse SI organoids were treated with 2-DG (5 mM) for 4 d followed by assessment of IAP activity (**C**). Expression of selective enterocyte markers was analyzed by qPCR; *n* = 3 biological repeats (**D**). **E**–**H** Human duodenum organoids were treated with 2-DG (5 mM) for 4 d and then analyzed for the expression of enterocyte markers (**E**) and the stem cell marker OLFM4 (**F**) by qPCR; *n* = 3 biological repeats. Representative image of organoid morphology following treatment with either 2-DG or control (**G**). Organoid numbers (*n* = 10 fields per group) (**H**, left panel)) and organoid size (*n* = 66 organoids per control group; *n* = 38 organoids per 2-DG treatment group) (**H**, right panel) were assessed. ^∗^*p* < 0.05; ^∗∗^*p* < 0.01; ^∗∗∗^*p* < 0.005. (**I**) Summary model illustrating the proposed role of BMP4/miR-181a-5p signaling pathway in intestinal cell proliferation and differentiation.

To next determine the role of HK1 in enterocyte differentiation, mouse SI organoids were infected with lentivirus expressing the HK1 shRNA or NTC shRNA and cultured in the presence of puromycin (2 μg/ml) to select for organoid cells with stable knockdown of HK1 as described [22]. The organoids with stable knockdown of HK1 or NTC shRNA were cultured for analysis of differentiation. As shown in Fig. 8, knockdown of HK1 increased enterocyte differentiation as noted by increased enterocyte IAP activity (Fig. 8A) and expression of the enterocyte markers KRT20, IAP, and VILLIN (Fig. 8B). To further demonstrate the role of HK1, we overexpressed HK1 in the human colon cancer cell line HT29. Treatment with sodium butyrate (NaBT), a histone deacetylase inhibitor which induces normal intestinal and cancer cell differentiation [20], induced enterocyte differentiation as demonstrated by dramatic increases in IAP activity and expression of enterocyte marker KRT20 expression in HT29 cells, these increases were significantly attenuated by overexpression of HK1 in HT29 cells (Supplementary Fig. 6).

To further determine the regulation of enterocyte maturation by HK1/glycolysis, we used the glycolysis inhibitor 2-deoxyglucose (2-DG). Similar to HK1 knockdown, treatment of mouse SI organoids with 2-DG increased enterocyte differentiation (Fig. 8C, D). Moreover, we show that 2-DG increased enterocyte differentiation (Fig. 8E), inhibited stem cell marker OLFM4 mRNA expression (Fig. 8F), and repressed organoid growth (Fig. 8G, H) in human IECs. These results suggest that the alteration of glycolysis by BMP4/miR-181a-5p mediates the effects of BMP4/miR-181a-5p on IEC proliferation and differentiation. Taken together, our results support a model in which BMP4/miR-181a-5p leads to glycolytic reprogramming and regulation of IEC proliferation and differentiation (Fig. 8I).

## Discussion

In this study, we identify the miR-181 family as downstream of BMP4 signaling in IECs. BMP4 repressed miR-181 family member expression, promoted enterocyte maturation, and inhibited IEC stemness. Consistent with these results, inhibition of miR-181a-5p increased enterocyte differentiation and inhibited mouse and human ISC renewal. BMP4/miR-181a-5p controls HK1/glycolysis which contributes to the maintenance of IEC stemness and enterocyte differentiation. Taken together, our results demonstrate a novel BMP4/miR-181a-5p/glycolysis signaling mechanism in the regulation of IEC proliferation and differentiation.

In this current study, we show that BMP4 represses miR-181 family in IECs. BMP signaling is a crucial regulator of intestinal proliferation and differentiation [11]. However, the molecular underpinnings of the BMP pathway in this context are unknown. BMP ligands bind to a type II receptor, which in turn bind and phosphorylate a type I receptor resulting in activation of the canonical BMP/SMAD signaling and enterocyte differentiation [38]. Activation of the BMP/SMAD signaling pathway promotes enterocyte differentiation via HNF4 and SMAD4 interaction [40]. In addition to SMAD-mediated signaling activation, BMPs can also activate noncanonical signaling including the activation of the MAPK pathway [41]. miRNAs have also been shown to modulate BMP signaling to regulate ISC proliferation [42]. For example, a stress-response miR-958 modulates BMP signaling and, in turn, ISC numbers during tissue regeneration [43]. miR-31 promotes ISC proliferation through repression of BMP signaling pathway [44]. BMP/SMAD signaling regulates miRNA expression at both the transcriptional and post-transcriptional level [45]; however, whether miRNAs are regulated by BMP4 in IECs is not known. We show that miR-181a-5p, as a downstream target of BMP4 signaling, mediates the function of BMP4 signaling in IECs. To dissect BMP4 signaling, we used a BMP type I receptor inhibitor LDN-193189. BMP4-mediated inhibition of miR-181a-5p and induction of enterocyte differentiation were attenuated by LDN-193189. LDN-193189 has been shown to affect all known BMP-induced signaling cascades [46]. Although LDN-193189 attenuated BMP4-mediated miR-181 repression in IECs, it remains to be defined whether BMP4 represses miR-181 through canonical BMP/SMAD signaling or noncanonical BMP signaling.

Accumulating evidence suggests that miR-181 plays important roles in the control of intestinal cell fate. For example, Jimenez et al. [14] reported that miR-181a is required for intestinal epithelial repair after mucosal injury. Our results demonstrate that miR-181a-5p is critical for the renewal of human and mouse ISCs suggesting a conserved role for miR-181a in the mammalian intestine. Indeed, miR-181a-5p is a conserved miRNA with the ability to regulate pathological processes, such as angiogenesis, inflammatory response, and obesity [47]. The expression of miR-181a in patients with ulcerative colitis was significantly lower than that in normal controls, and miR-181a deficiency promotes development of severe colonic inflammation in DSS-induced colitis in mice [14, 48]. These results indicate that miR-181a may play an important role in the pathogenesis of IBD. In our study, we identified miR-181 as the target of BMP4 in IECs. In line with these findings, excessive BMP4 in the intestinal crypt–villus axis impairs, whereas transgenic overexpression of the BMP4 antagonist Grem1 enhances the intestinal regeneration in DSS-induced colitis [49]. Together, these findings further suggest an important role for miR-181 in mediating the effects of BMP4 on intestinal inflammation and regeneration. However, whether administration of mir-181a-5p attenuates intestinal inflammation remains to be defined.

The intestinal epithelium lacking Dicer, a key enzyme in the miRNA biogenesis pathway, shows dysregulation of differentiation and impaired intestinal barrier function [50]; however, whether individual miRNAs directly regulate IEC differentiation remains poorly understood. Our results demonstrate that miR-181a-5p is an important regulator of ISC differentiation since miR-181a-5p inhibition enhances enterocyte maturation. The wide-ranging effects of the miR-181 family members on regulating differentiation have been demonstrated in multiple tissue types [51]. Moreover, the inhibitory function of miR-181 family members on differentiation has been shown in osteoclasts ([52]). In contrast, miR-181 family members demonstrate a pro-differentiation effect for a number of cell lineages including osteoblasts, hypertrophic chondrocytes, adipocytes, myoblasts, B cells, NK/NKT cells, and thymocytes [51, 53]. This differential effect of miR-181 is likely due to specific cell types expressing distinct populations of mRNAs targeted by miR-181 homologs.

We found that BMP4/miR-181a-5p regulates glycolysis in IECs. BMP4 significantly represses the expression of the miR-181 family in IECs, and this repression of miR-181a-5p decreases glycolysis. In agreement with our findings, treatment with BMP4 decreased glycolytic capacity in T cells [54]. miR-181a-5p has been shown to reduce the electron transport chain, resulting in an increase in HK2 and glycolytic activity in liver cancer cells [55]. In addition, miR-181a-5p promoted glycolysis by targeting N-myc downstream-regulated gene 2 in breast cancer cells [56]. Although we show that inhibition of miR-181a-5p represses HK1 expression and glycolysis, the mechanisms involved in miR-181a-5p regulation of HK1/glycolysis remain to be further defined. Previously, we have shown HK2-dependent glycolytic activity plays a critical role in the maintenance of ISC renewal and differentiation [19]. Taken together, the results from our current study suggest that BMP4/miR-181a-5p regulates enterocyte differentiation and IEC proliferation by the regulation of glycolysis.

In conclusion, our results demonstrate that miR-181 expression is regulated in IECs by the BMP4 signaling pathway. Importantly, these novel findings add the miR-181 family to the growing list of factors regulated by BMP4. Moreover, our results provide a better understanding of the potential mechanism underlying the role of the BMP4 pathway in intestinal cell homeostasis.

## Supplementary information

Supplementary Figure 1

Supplementary Figure 2

Supplementary Figure 3

Supplementary Figure 4

Supplementary Figure 5

Supplementary Figure 6

Supplemental Figure Legends & Supplemental Table 1 & Supplemental Materials, Methods, and References

Original Blots

## Data Availability

The miRNA-seq and total RNA-seq data reported in this study is available from Gene Expression Omnibus (GEO) with accession code GSE266339 and GSE294782, respectively. All other data analyzed during this study are included in this article and the supplemental data files.
