# Supplementary figures and images for: miR-181a-5p mediates the effects of BMP4 on intestinal cell proliferation and differentiation
Source: Cell Death Dis. 2025 May 28;16(1):420. doi: 10.1038/s41419-025-07730-w (PMC12120108; doi:10.1038/s41419-025-07730-w)

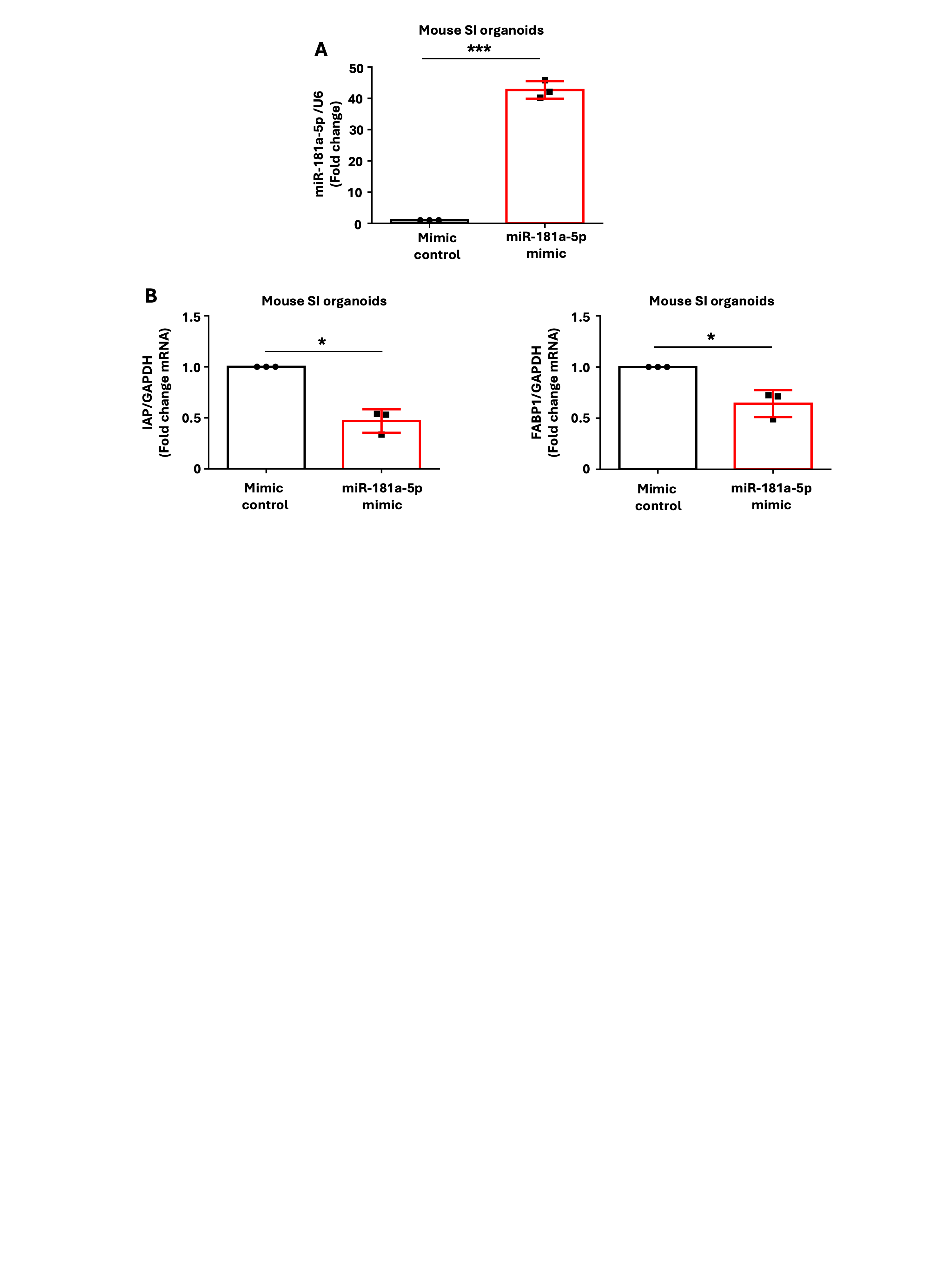

Supplement: Supplementary file 1 — Supplementary Figure 1 [file 41419_2025_7730_MOESM1_ESM.tif]

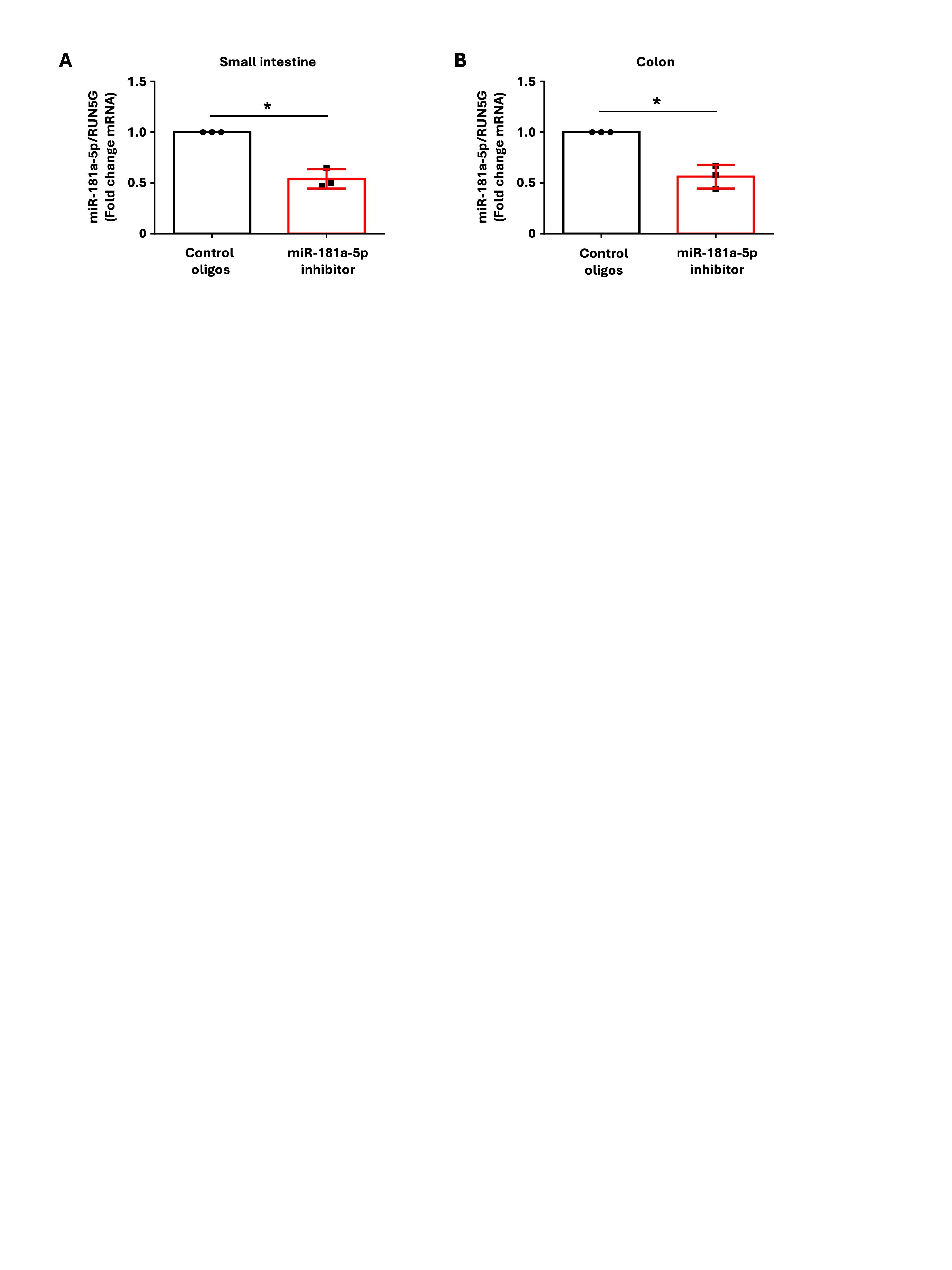

Supplement: Supplementary file 2 — Supplementary Figure 2 [file 41419_2025_7730_MOESM2_ESM.tif]

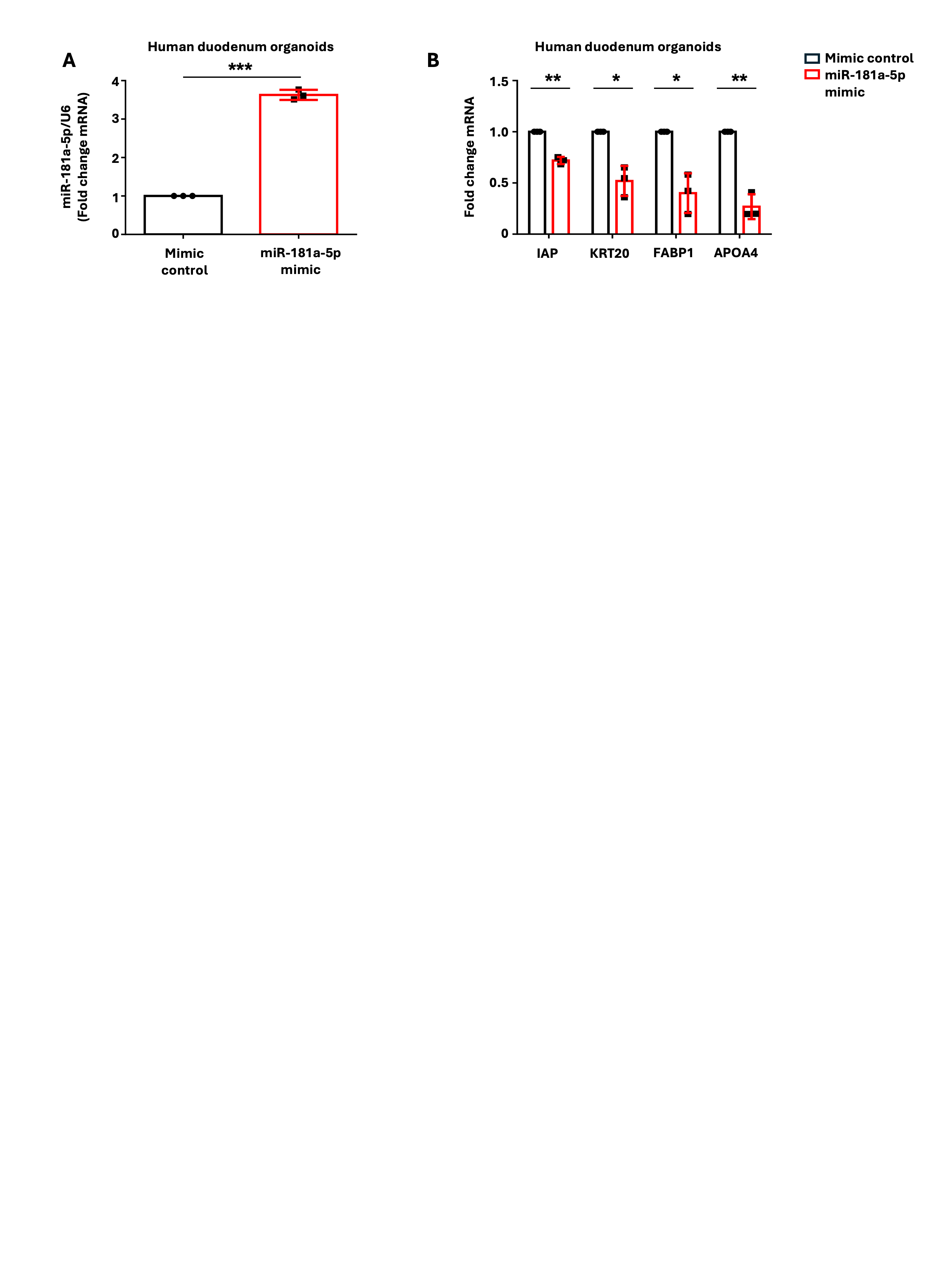

Supplement: Supplementary file 3 — Supplementary Figure 3 [file 41419_2025_7730_MOESM3_ESM.tif]

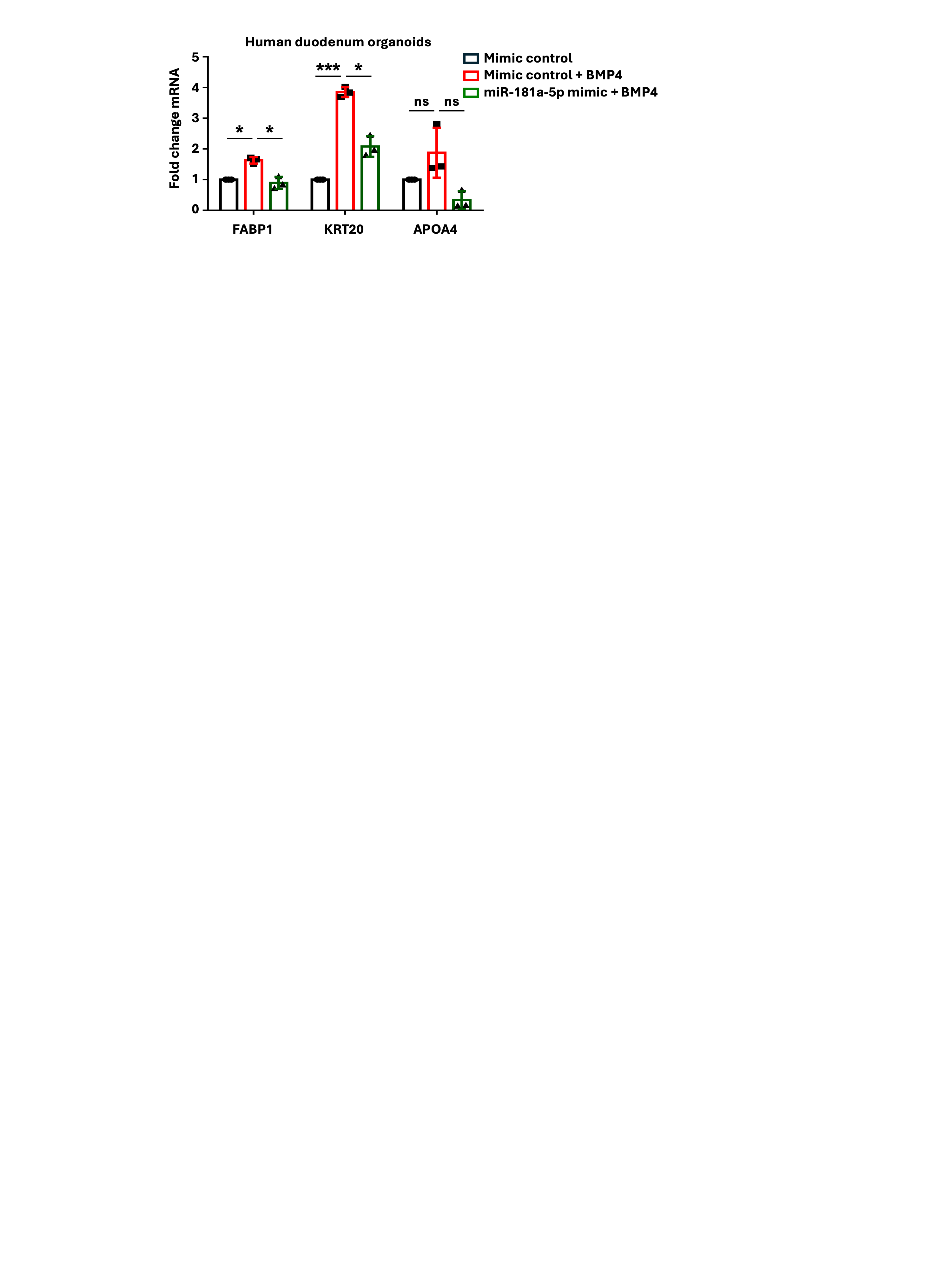

Supplement: Supplementary file 4 — Supplementary Figure 4 [file 41419_2025_7730_MOESM4_ESM.tif]

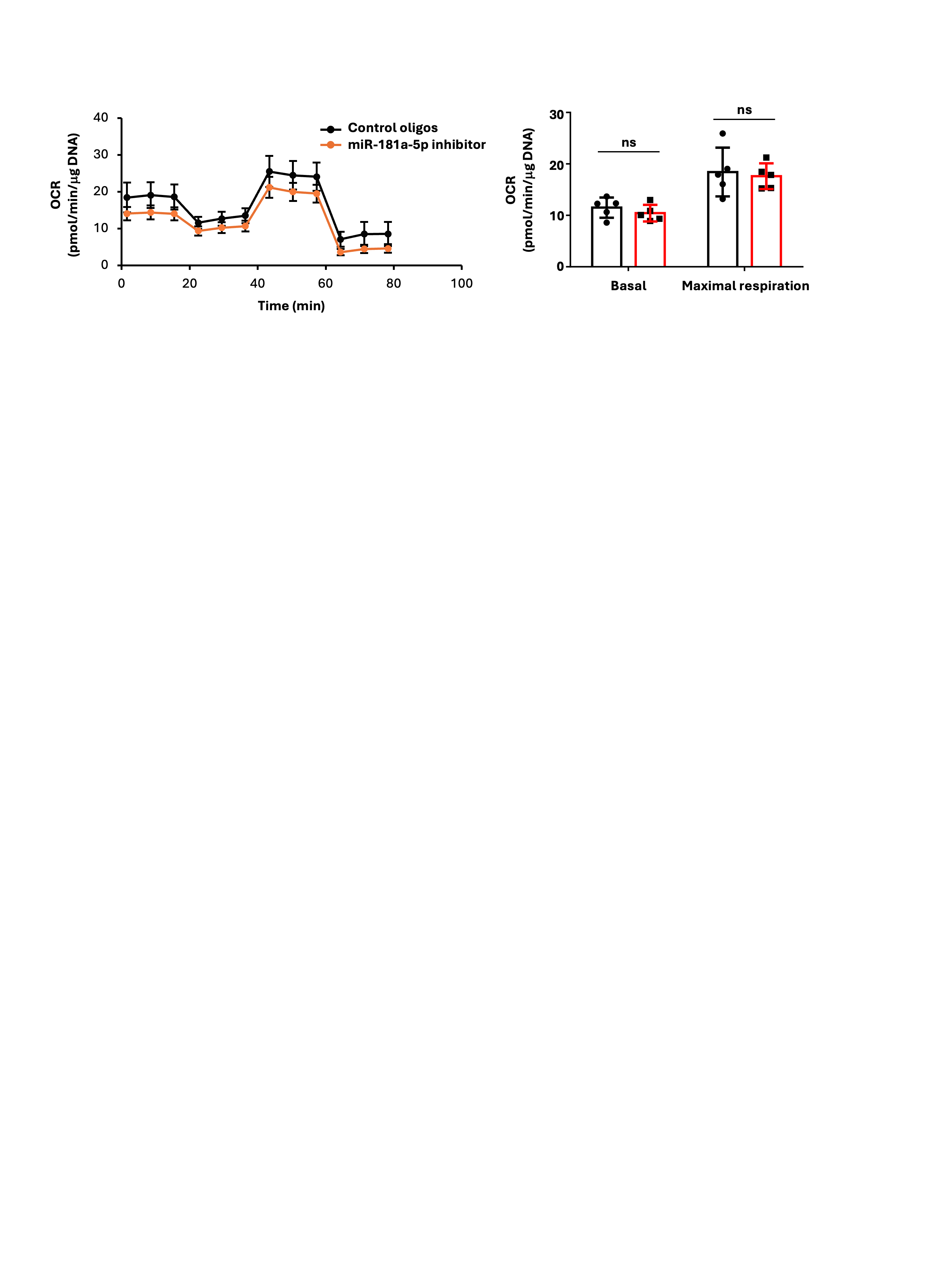

Supplement: Supplementary file 5 — Supplementary Figure 5 [file 41419_2025_7730_MOESM5_ESM.tif]

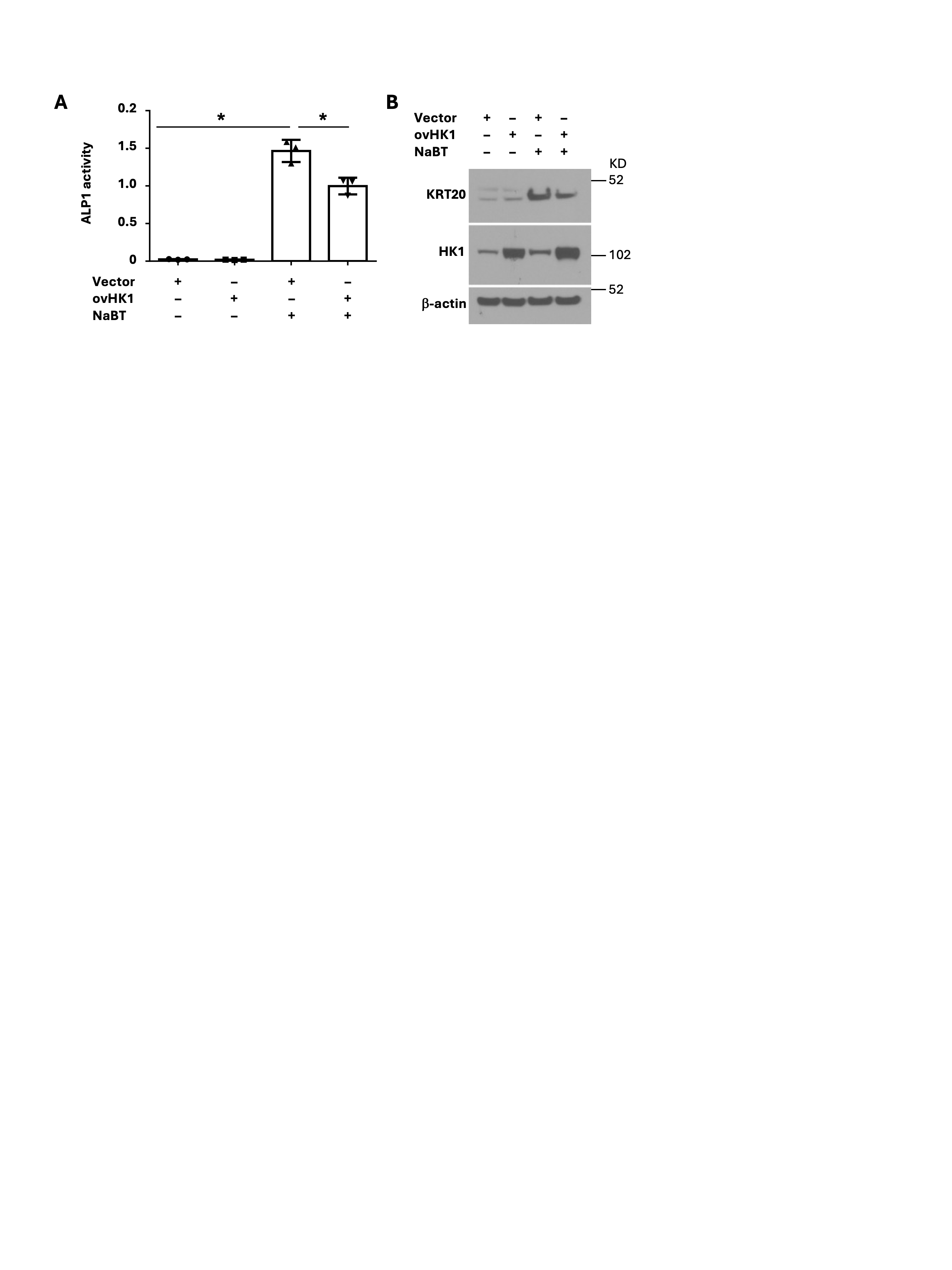

Supplement: Supplementary file 6 — Supplementary Figure 6 [file 41419_2025_7730_MOESM6_ESM.tif]
