# Supplementary material for: miR-181a-5p mediates the effects of BMP4 on intestinal cell proliferation and differentiation
Source: Cell Death Dis. 2025 May 28;16(1):420. doi: 10.1038/s41419-025-07730-w (PMC12120108; doi:10.1038/s41419-025-07730-w)
Supplement: Supplementary file 7 — Supplemental Figure Legends & Supplemental Table 1 & Supplemental Materials, Methods, and References [file 41419_2025_7730_MOESM7_ESM.docx]

**Supplemental Figure 1.** **miR-181a-5p mimics decreased enterocyte marker expression in mouse SI organoids.** Mouse SI organoids with overexpression of miR-181a-5p mimic or mimic control were assessed for the expression of miR-181a-5p (**A**) or selective enterocyte markers (**B**) by qPCR; *n* = 3 biological repeats. ^∗^*P*<.05; ****P*<.005.

**Supplemental Figure 2. A cell-permeable miR-181a-5p inhibitor is effective in the intestine 3 days after one dose.** Mice were IP injected with control oligos or miR-181a-5p inhibitor (20 mg/kg). Three d after injection, SI (**A**) and colonic (**B**) mucosa was collected and total RNA extracted and the expression of miR-181a-5p was analyzed by qPCR. *n* = 3 mice per group. **p* < .05.

**Supplemental Figure 3. Overexpression of miR-181a-5p represses the expression of enterocyte differentiation markers in human duodenum organoids.** Human duodenum organoids infected with lentivirus expressing miR-181a-5p mimic or mimic control were assessed for the expression of miR-181a-5p (**A**) or enterocyte differentiation markers (**B**) by qPCR; *n* = 3 biological repeats. **p* <.05; ***p* <.01; ****p* <.005.

**Supplemental Figure 4. Overexpression of miR-181a-5p attenuates BMP4-induced expression of enterocyte differentiation markers in human duodenum organoids.** Human duodenum organoids infected with lentivirus expressing miR-181a-5p mimic or control mimic were treated with or without BMP4 (100 ng/ml) for 5 d. qPCR analysis of selected enterocyte markers; *n* = 3 biological repeats. **p* <.05; ****p* <.005.

**Supplemental Figure 5. Treatment with LNA miR-181a-5p inhibitor has minor effect on OCR.** Mouse SI organoids were treated with LNA miR-181a-5p inhibitor or control oligos (0.75 μM) for 4 d followed by Seahorse analysis for oxygen consumption. The normalized OCR was shown. (control oligos *n* = 4 biological repeats; miR-181a-5p *n* = 5 biological repeats).

**Supplemental Figure 6. Overexpression of HK1 inhibits the expression of enterocyte differentiation markers in HT29 cells.** HT29 cells with overexpression of HK1 were treated with NaBT (5 mM) for 48 h. (**A**) IAP activity assay. *n* = 3 biological repeats. **p* <.05. (**B**) Western blotting analysis.

| **gene_symbol** | **logFC** | **logCPM** | **PValue** | **FDR** |
| --- | --- | --- | --- | --- |
| **Vil1** | -0.09472 | 9.228135 | 0.514436 | 0.761542 |
| **Apoa4** | -0.55976 | 8.91107 | 0.045644 | 0.201385 |
| **Anpep** | -2.14367 | 9.891145 | 1.48E-17 | 7.06E-15 |
| **Cbr1** | -0.00954 | 7.124634 | 0.95018 | 0.993073 |
| **Fabp1** | -0.91366 | 8.62968 | 0.00195 | 0.024968 |
| **Alpi** | -0.70794 | 5.915054 | 0.156366 | 0.411397 |

**Supplemental Table 1.** RNA-seq analysis showing decreased expression of enterocyte markers in mouse SI organoids overexpressing miR-181-5p mimic.

# **Supplemental Materials and Methods**

**HT29 stable cells expression HK1.** The Full-length sequence of human HK1 was amplified from the plasmid expressing human HK1 (#21917, Addgene, Watertown, MA) and was sub-cloned into the pCW57-GFP-2A-MCS lentiviral vector (#41393, Addgene). The human CRC cell line, HT29, purchased from ATCC, was maintained in McCoy’s 5A supplemented with 10% fetal calf serum (FCS). HT29 cells were tested for Mycoplasma contamination using a sensitive PCR-based Mycoplasma detection kit (Biovision) and were found to be negative. Authentications were confirmed by a 100% match in comparison to the reference STR profiles from ATCC.  HT29 cells were infected with lentiviral particles containing protein expression vector encoding gene for HK1, selected with puromycin (5 μg/ml), and induced by doxycycline (400 ng/ml) as described ^1^.

**Total RNA-seq.** The total RNA from control miRNA or miR-181a-5p mimic overexpression mice organoids were collected and total RNA-seq was performed as described ^2^. Briefly, total RNA was quantified by automated electrophoresis on the 2100 Bioanalyzer [Agilent Technologies, Inc., Santa Clara, CA] and normalized to 250 ng total RNA input in 10 uL. Ribosomal RNA was then depleted using Roche KAPA RiboErase (HMR) enzymatic depletion kit. Following ribosomal depletion and subsequent purification, RNA was fragmented, and cDNA synthesized. Total RNAseq libraries were then constructed using the Roche KAPA RNA HyperPrep kit and sequenced using a Single-read 100 cycle kit on the HiSeq 2500 [Illumina, Inc., San Diego, CA]. Sequencing reads were trimmed and filtered using Trimmomatic ^3^ to remove adapters and low-quality bases. Trimmed reads were mapped to the mouse reference genome assembly GRCm38 (mm10) transcripts annotation using RSEM ^4^. RSEM results normalization and differential expression analysis were performed using the R package EdgeR ^5^. Significantly up/downregulated genes between groups were determined as fold change >= 2 and q-value < 0.05.

**Supplementary References**

1. Li, C. *et al.* Regulation of SIRT2 by Wnt/β-catenin signaling pathway in colorectal cancer cells. *Biochim Biophys Acta Mol Cell Res* **1868**, 118966 (2021).

2. Wei, R. *et al.* Ketogenesis Attenuates KLF5-Dependent Production of CXCL12 to Overcome the Immunosuppressive Tumor Microenvironment in Colorectal Cancer. *Cancer Res* **82**, 1575-1588 (2022).

3. Bolger, A.M., Lohse, M. & Usadel, B. Trimmomatic: a flexible trimmer for Illumina sequence data. *Bioinformatics* **30**, 2114-2120 (2014).

4. Li, B. & Dewey, C.N. RSEM: accurate transcript quantification from RNA-Seq data with or without a reference genome. *BMC Bioinformatics* **12**, 323 (2011).

5. Love, M.I., Huber, W. & Anders, S. Moderated estimation of fold change and dispersion for RNA-seq data with DESeq2. *Genome Biol* **15**, 550 (2014).
