# Supplementary material for: miR-181a-5p mediates the effects of BMP4 on intestinal cell proliferation and differentiation
Source: Cell Death Dis. 2025 May 28;16(1):420. doi: 10.1038/s41419-025-07730-w (PMC12120108; doi:10.1038/s41419-025-07730-w)
Supplement: Supplementary file 8 — Original Blots [file 41419_2025_7730_MOESM8_ESM.pptx]

## Slide 1
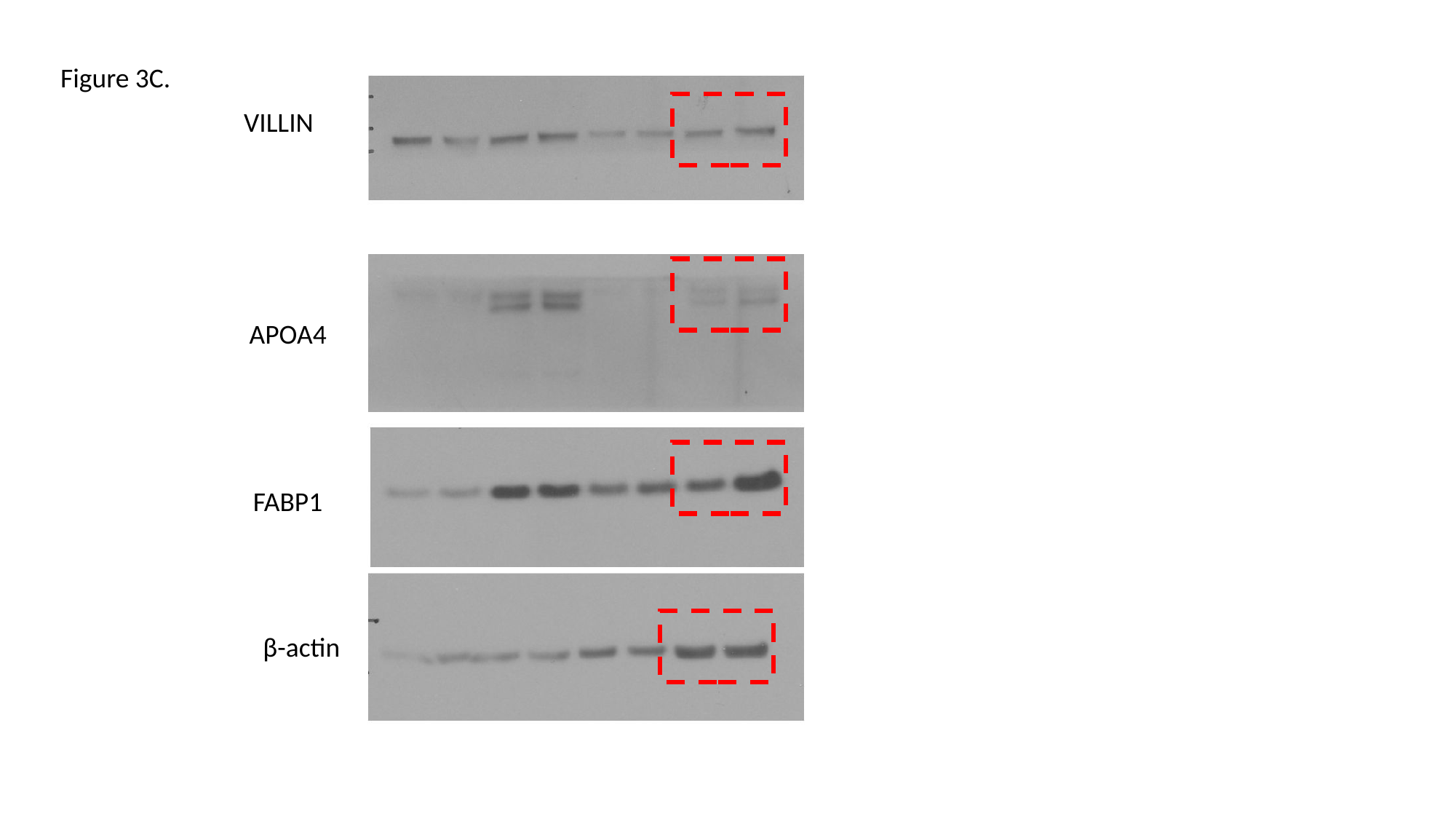

Figure 3C.
VILLIN
APOA4
FABP1
β-actin

## Slide 2
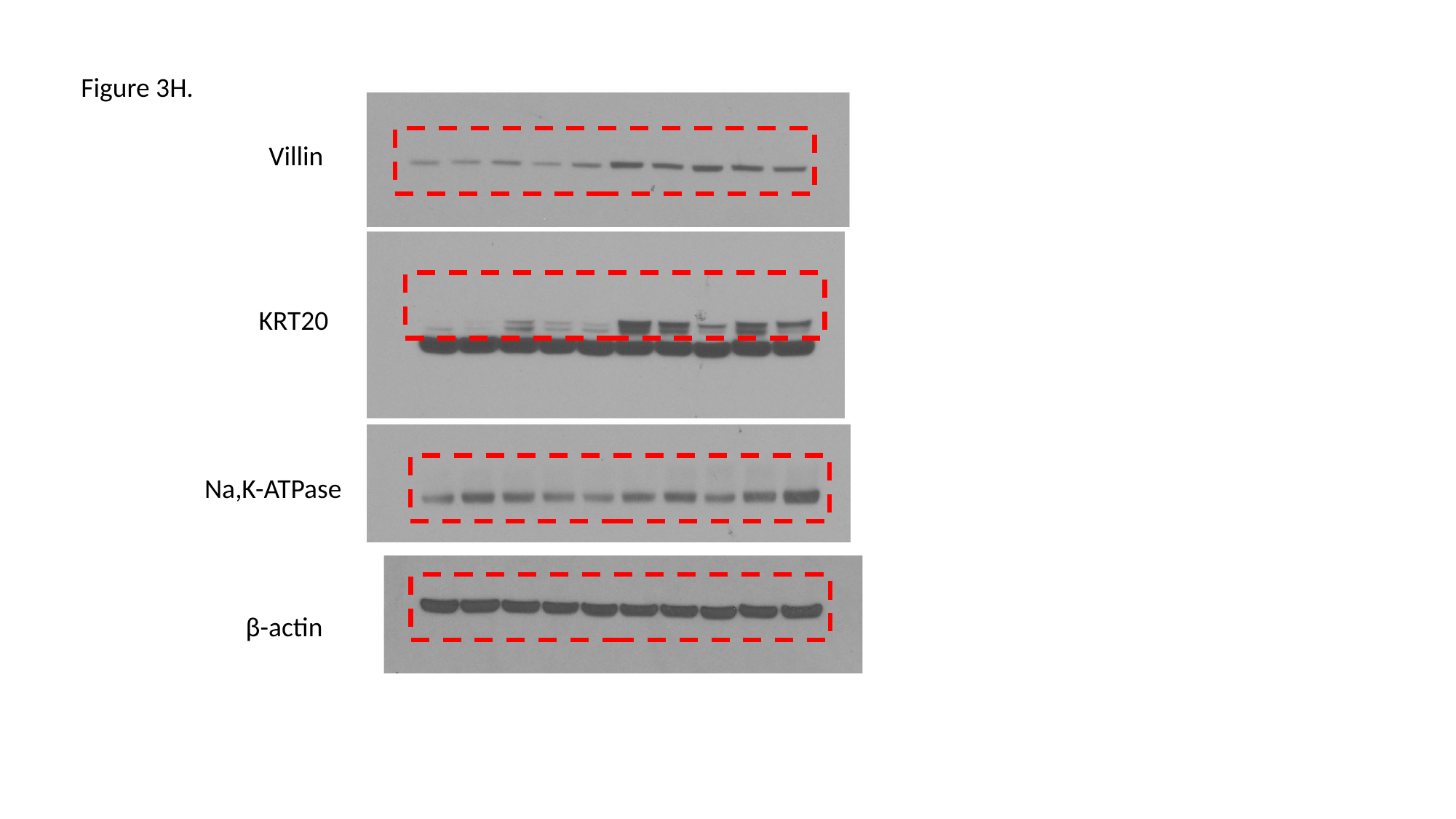

Figure 3H.
Villin
KRT20
Na,K-ATPase
β-actin

## Slide 3
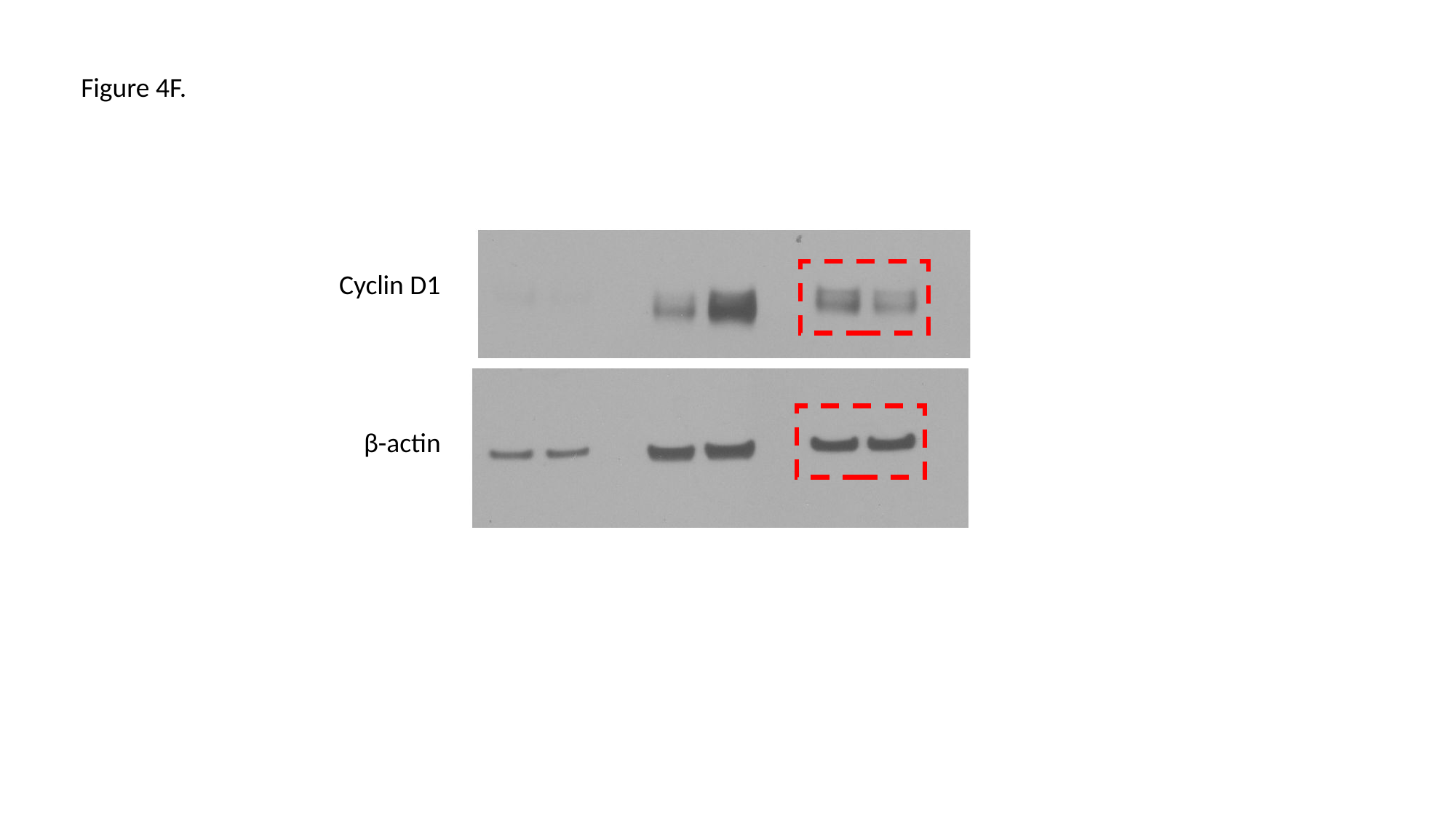

Figure 4F.
Cyclin D1
β-actin

## Slide 4
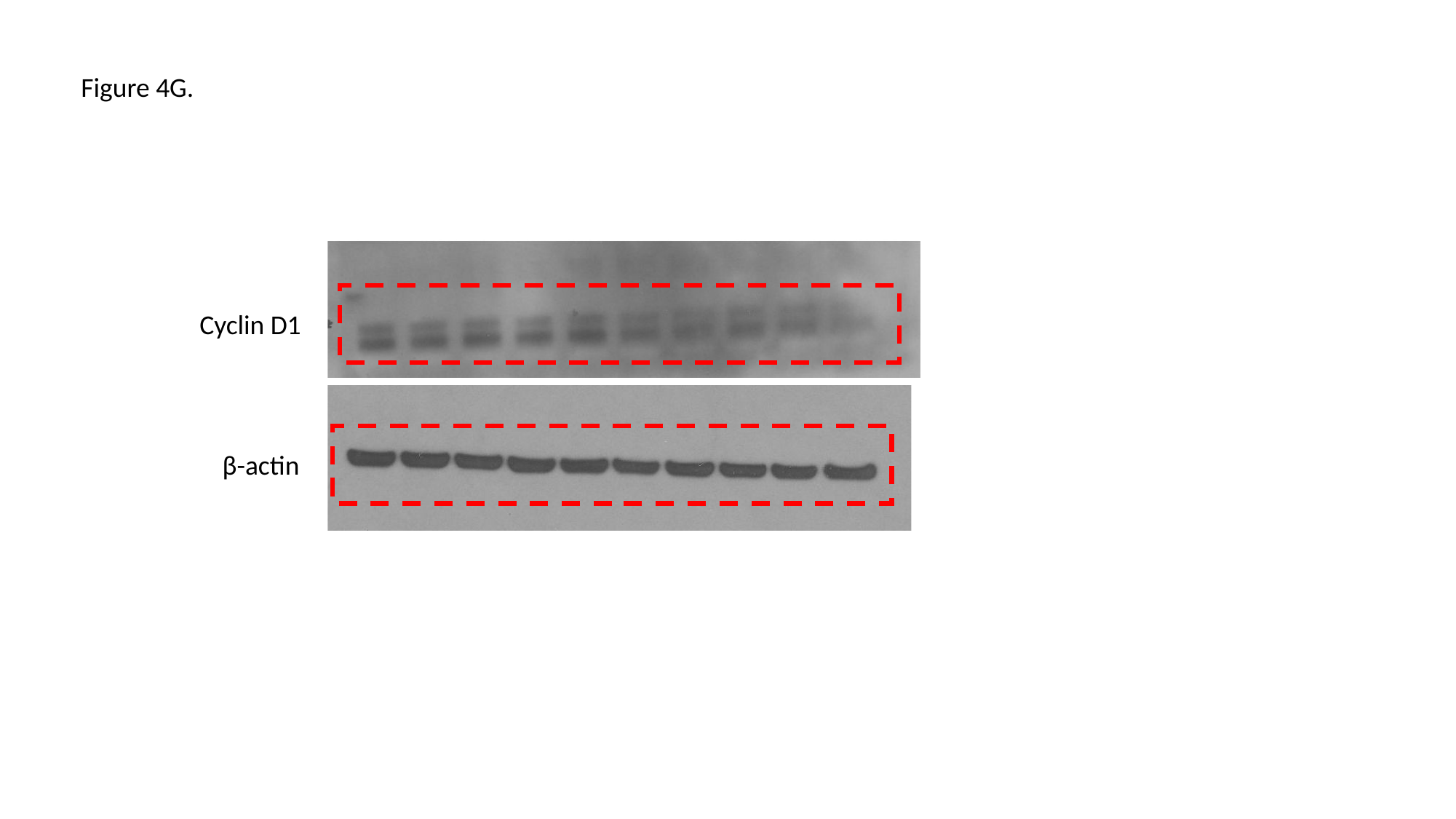

Figure 4G.
Cyclin D1
β-actin

## Slide 5
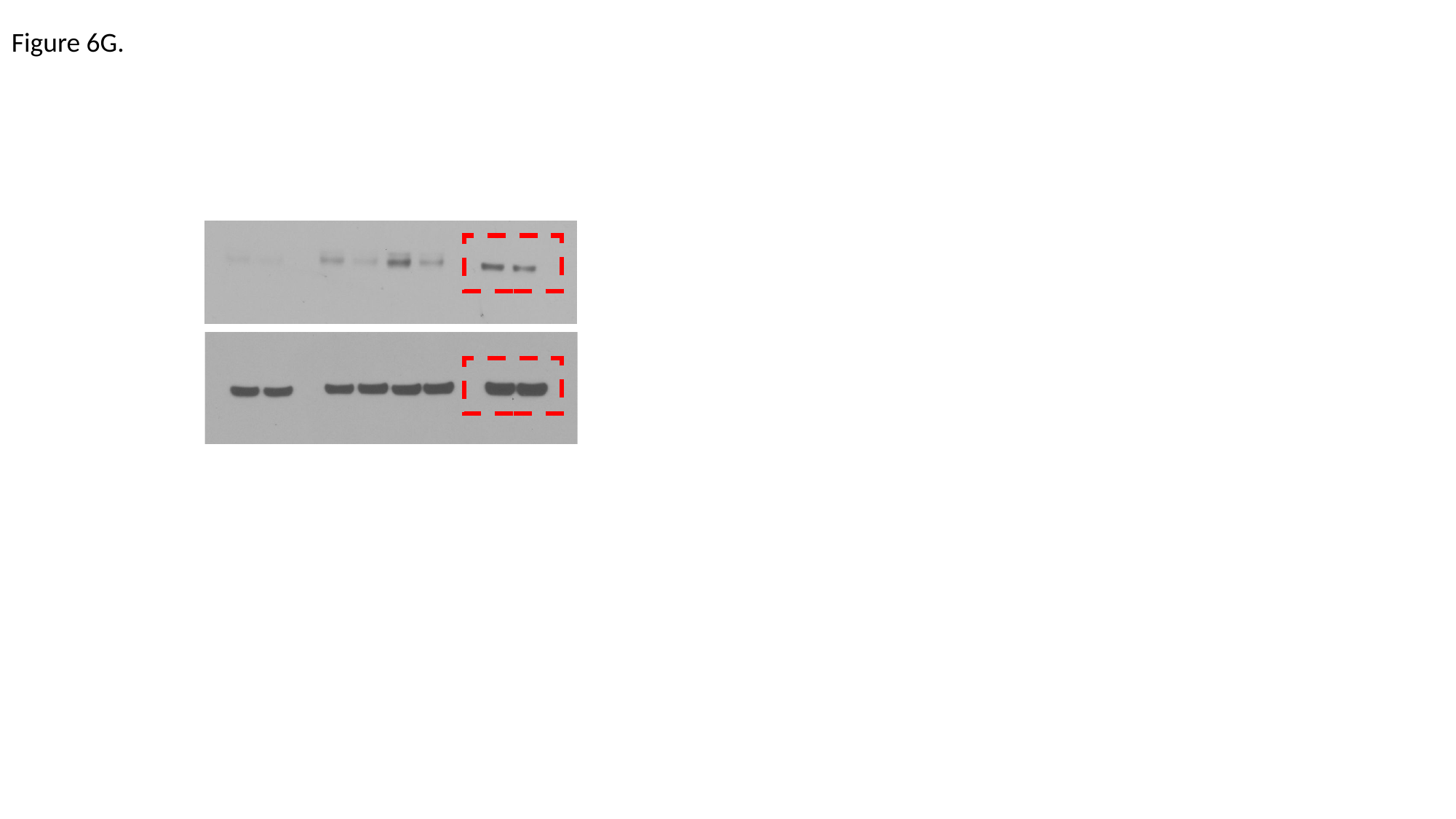

Figure 6G.

## Slide 6
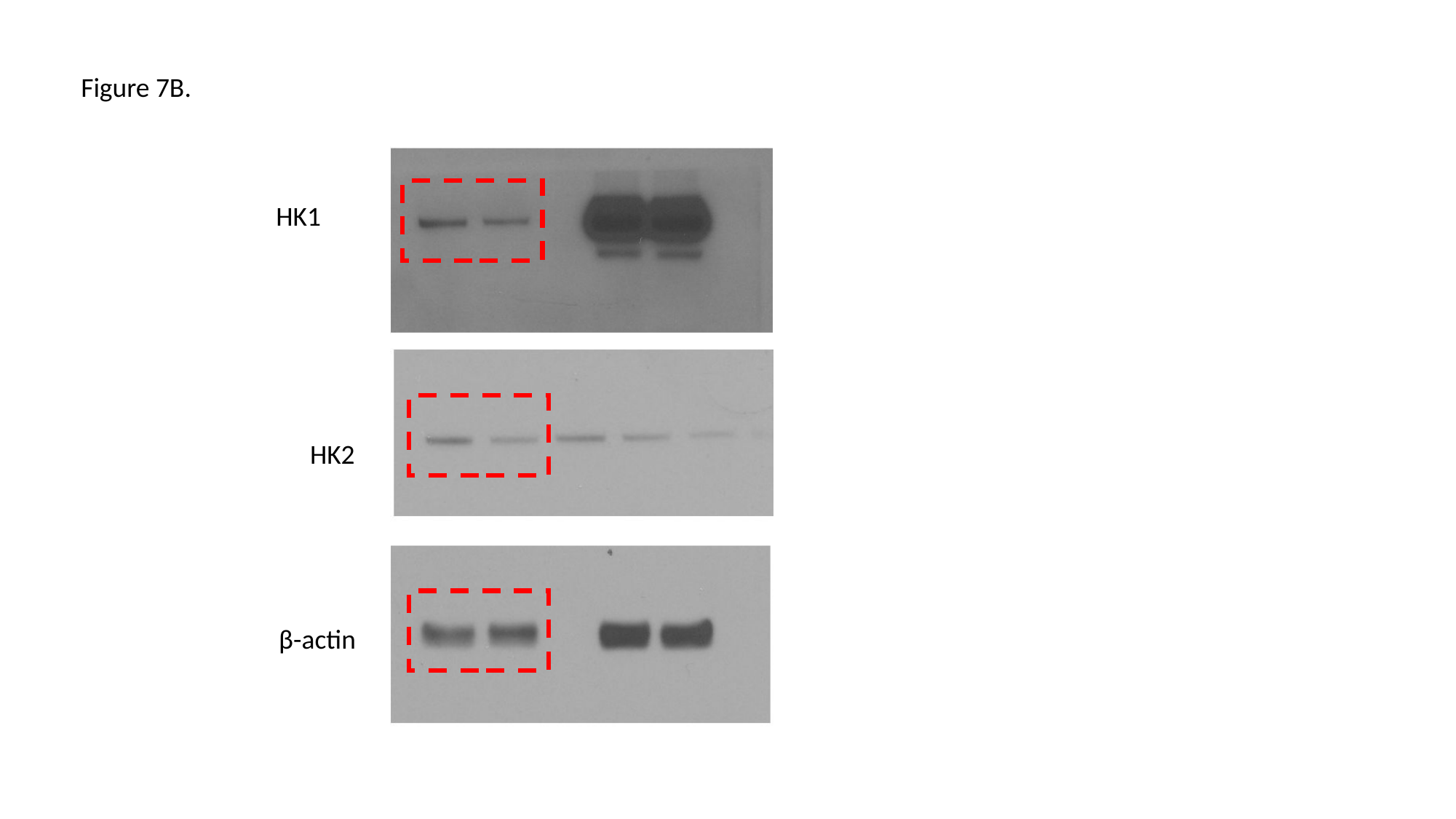

Figure 7B.
HK1
HK2
β-actin

## Slide 7
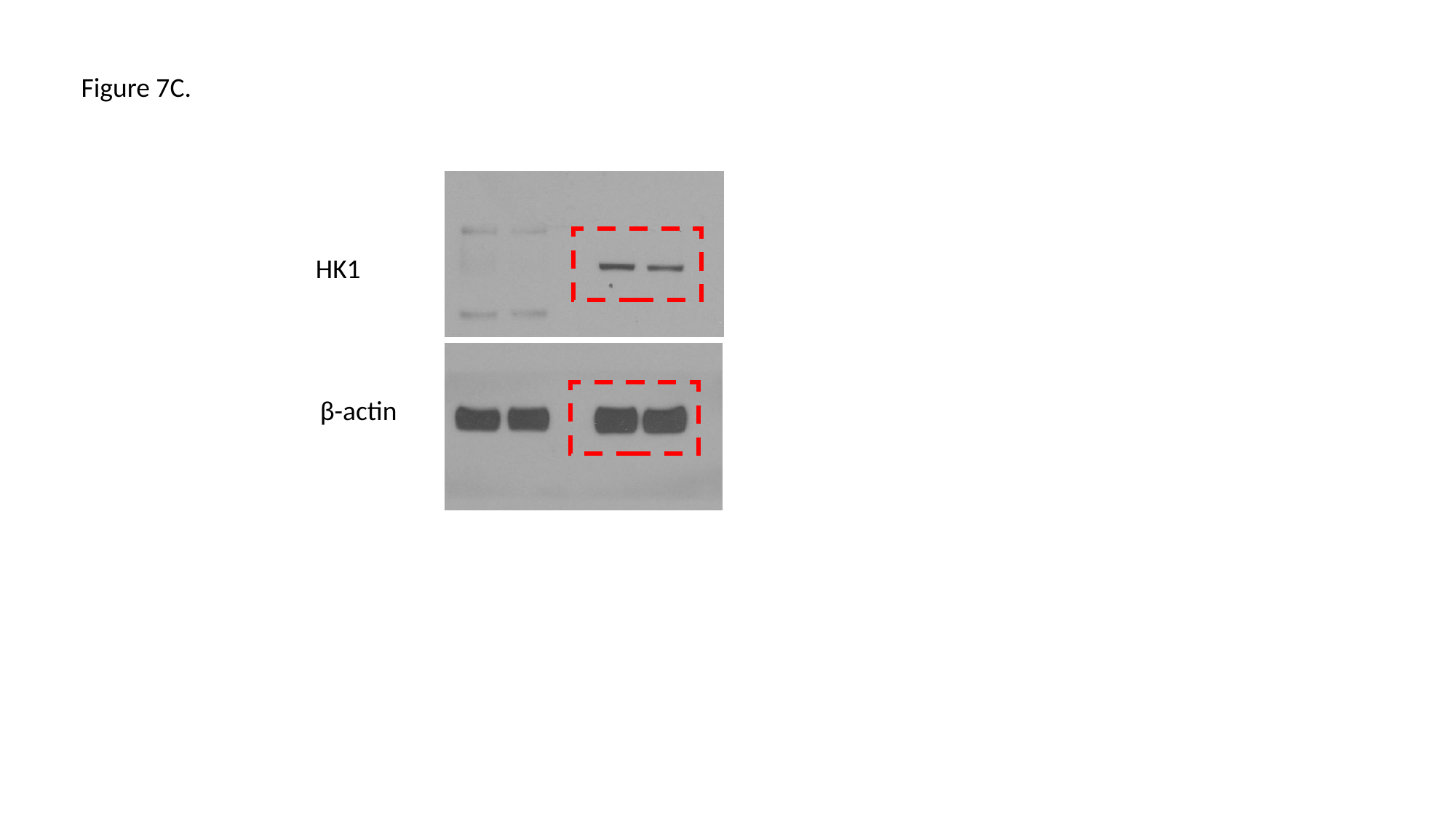

Figure 7C.
HK1
β-actin

## Slide 8
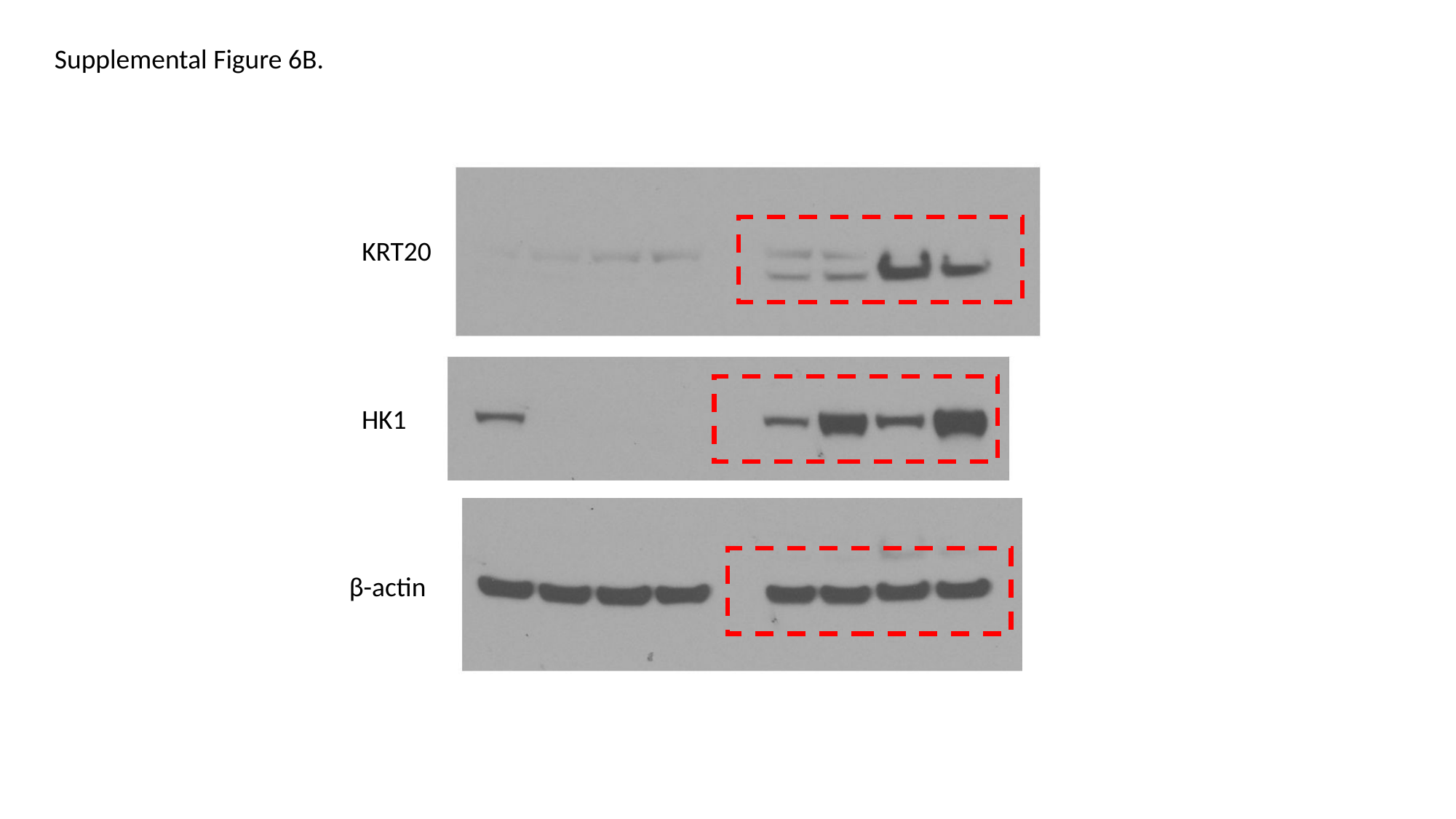

Supplemental Figure 6B.
KRT20
HK1
β-actin
